# TOPORS-mediated RAD51 SUMOylation facilitates homologous recombination repair

**DOI:** 10.1093/nar/gkac009

**Published:** 2022-01-21

**Authors:** Gurusamy Hariharasudhan, Seo-Yeon Jeong, Min-Ji Kim, Sung Mi Jung, Gwanwoo Seo, Ju-Ran Moon, Sumi Lee, In-Youb Chang, Younghoon Kee, Ho Jin You, Jung-Hee Lee

**Affiliations:** Laboratory of Genomic Instability and Cancer Therapeutics, Chosun University School of Medicine, 309 Pilmun-daero, Dong-gu, Gwangju 61452, Republic of Korea; Department of Cellular and Molecular Medicine, Chosun University School of Medicine, 309 Pilmun-daero, Dong-gu, Gwangju 61452, Republic of Korea; Laboratory of Genomic Instability and Cancer Therapeutics, Chosun University School of Medicine, 309 Pilmun-daero, Dong-gu, Gwangju 61452, Republic of Korea; Department of Pharmacology, Chosun University School of Medicine, 309 Pilmun-daero, Dong-gu, Gwangju 61452, Republic of Korea; Laboratory of Genomic Instability and Cancer Therapeutics, Chosun University School of Medicine, 309 Pilmun-daero, Dong-gu, Gwangju 61452, Republic of Korea; Department of Cellular and Molecular Medicine, Chosun University School of Medicine, 309 Pilmun-daero, Dong-gu, Gwangju 61452, Republic of Korea; Laboratory of Genomic Instability and Cancer Therapeutics, Chosun University School of Medicine, 309 Pilmun-daero, Dong-gu, Gwangju 61452, Republic of Korea; Department of Cellular and Molecular Medicine, Chosun University School of Medicine, 309 Pilmun-daero, Dong-gu, Gwangju 61452, Republic of Korea; Laboratory of Genomic Instability and Cancer Therapeutics, Chosun University School of Medicine, 309 Pilmun-daero, Dong-gu, Gwangju 61452, Republic of Korea; Department of Pharmacology, Chosun University School of Medicine, 309 Pilmun-daero, Dong-gu, Gwangju 61452, Republic of Korea; Laboratory of Genomic Instability and Cancer Therapeutics, Chosun University School of Medicine, 309 Pilmun-daero, Dong-gu, Gwangju 61452, Republic of Korea; Department of Cellular and Molecular Medicine, Chosun University School of Medicine, 309 Pilmun-daero, Dong-gu, Gwangju 61452, Republic of Korea; Laboratory of Genomic Instability and Cancer Therapeutics, Chosun University School of Medicine, 309 Pilmun-daero, Dong-gu, Gwangju 61452, Republic of Korea; Department of Cellular and Molecular Medicine, Chosun University School of Medicine, 309 Pilmun-daero, Dong-gu, Gwangju 61452, Republic of Korea; Department of Anatomy, Chosun University School of Medicine, 309 Pilmun-daero, Dong-gu, Gwangju 61452, Republic of Korea; Department of New Biology, Daegu Gyeongbuk Institute of Science and Technology (DGIST), 333 Techno-Joongang-daero, Dalseong-gun, Daegu 42988, Republic of Korea; Laboratory of Genomic Instability and Cancer Therapeutics, Chosun University School of Medicine, 309 Pilmun-daero, Dong-gu, Gwangju 61452, Republic of Korea; Department of Pharmacology, Chosun University School of Medicine, 309 Pilmun-daero, Dong-gu, Gwangju 61452, Republic of Korea; Laboratory of Genomic Instability and Cancer Therapeutics, Chosun University School of Medicine, 309 Pilmun-daero, Dong-gu, Gwangju 61452, Republic of Korea; Department of Cellular and Molecular Medicine, Chosun University School of Medicine, 309 Pilmun-daero, Dong-gu, Gwangju 61452, Republic of Korea

## Abstract

Homologous recombination (HR) is critical for error-free repair of DNA double-strand breaks. Chromatin loading of RAD51, a key protein that mediates the recombination, is a crucial step in the execution of the HR repair. Here, we present evidence that SUMOylation of RAD51 is crucial for the RAD51 recruitment to chromatin and HR repair. We found that topoisomerase 1-binding arginine/serine-rich protein (TOPORS) induces the SUMOylation of RAD51 at lysine residues 57 and 70 in response to DNA damaging agents. The SUMOylation was facilitated by an ATM-induced phosphorylation of TOPORS at threonine 515 upon DNA damage. Knockdown of TOPORS or expression of SUMOylation-deficient RAD51 mutants caused reduction in supporting normal RAD51 functions during the HR repair, suggesting the physiological importance of the modification. We found that the SUMOylation-deficient RAD51 reduces the association with its crucial binding partner BRCA2, explaining its deficiency in supporting the HR repair. These findings altogether demonstrate a crucial role for TOPORS-mediated RAD51 SUMOylation in promoting HR repair and genomic maintenance.

## INTRODUCTION

Accurate repair of DNA double-strand breaks (DSBs) that result from endogenous and exogenous damage is critical for maintaining chromosomal integrity. Repair via homologous recombination (HR) requires an intact homologous sister chromatid as a template to ensure that repair occurs in an error-free manner (1–4). RAD51 is a key component of the HR repair that plays a critical role in the response to both DSBs and stalled replication forks (5–11). The first step in the HR repair is nucleolytic degradation at DSB ends to yield 3′ single-stranded DNA (ssDNA) overhangs, which are coated with replication protein A (RPA) and then replaced with RAD51 to form a nucleoprotein filament, triggering the exchange of DNA homologs. RAD51 localization and activation are regulated by additional factors, most importantly BRCA2, which stimulates the binding of RAD51 to ssDNA (12–15).

In addition to its role in HR repair, RAD51 plays a separate role in replication stress (16). It stabilizes replication fork intermediates and prevents degradation of newly synthesized DNA at stalled replication forks, thereby facilitating replication fork restart (10,17). This fork protection role depends upon BRCA2, which facilitates loading of RAD51 onto replication forks and, in turn, prevents the effects of MRE11-mediated fork degradation (18,19). In addition, RAD51 promotes efficient reversal of stalled replication forks (8,20), which is critical for maintaining chromosome stability in BRCA2-defective cells (21).

Posttranslational modification (PTM) is a dynamic and critical way to regulate HR. After RAD51 is phosphorylated by PLK1 or CK2, it accumulates at DSB sites and facilitates HR (22,23). RAD51 is negatively regulated during replication stress through ubiquitination by FBH1 (24). It has also been shown that polyubiquitination of RAD51 by RFWD3 facilitates its timely removal from sites of DNA damage to allow downstream steps to proceed and complete the HR repair (25). Polyubiquitination of RAD51 also appears to inhibit RAD51 from associating with BRCA2, as deubiquitination of RAD51 by UCHL3 promotes the RAD51–BRCA2 association and facilitates HR (26). In addition, SUMOylation of RPA70 and BLM is necessary for interactions between these proteins and RAD51, and modulates recruitment of RAD51 to repair sites (27,28). Moreover, SUMOylated MDC1 and Rad52 influence the loading of RAD51 to DNA lesions (29–32). However, although a relationship between SUMOylation and RAD51 has been extensively studied (27,33–35), the question of whether RAD51 is directly SUMOylated in response to DNA damage remained unanswered. Because RAD51 is a central player in HR, it is crucial to understand how it might be SUMOylated in response to DNA damage and to elucidate the functional relevance of this modification.

In the present study, we demonstrate that the SUMO E3 ligase activity of topoisomerase 1-binding arginine/serine-rich protein (TOPORS) catalyzes the SUMOylation of RAD51 both *in vitro* and *in vivo*. In addition, we show that SUMOylation is a critical modulation of RAD51 that affects its recruitment to DNA lesions and promotes HR-mediated DSB repair.

## MATERIALS AND METHODS

### Cell culture

U2OS, HeLa, HEK293T, TOPORS^+/+^ and TOPORS^−/−^ MEFs were cultured in Dulbecco’s modified Eagle’s medium (Invitrogen, USA) supplemented with 10% heat-inactivated fetal bovine serum (Invitrogen), 100 units/ml penicillin and 100 μg/ml streptomycin (Invitrogen). Cells were maintained in a humidified incubator containing 5% CO_2_ at 37°C. All cells were obtained from the American Type Culture Collection (Manassas, VA, USA). TOPORS^+/+^ and TOPORS^−/−^ mouse embryonic fibroblast (MEF) cells were obtained from Dr Eric H. Rubin (36). To induce DNA damage, exponentially growing cells were treated with ^137^Cs γ-ray at a dose of 2 or 5 Gy (Gammacell 3000 Elan; Best Theratronics, Ottawa, Canada), 2 mM hydroxyurea (HU, Sigma-Aldrich, USA) and then allowed to recover at 37°C for indicated time points.

### Antibodies

The antibodies used for immunoblot, immunoprecipitation and immunofluorescence analysis are provided in Supplementary Table S1.

### siRNA transfection and generation of stable TOPORS knockdown cells

For the knockdown of RAD51 and TOPORS, cells were transiently transfected with siRNA using Lipofectamine RNAiMax (Invitrogen) according to the manufacturer’s instructions. The siRNA target sequences were as follows: RAD51 siRNA, 5′-CUA AUC AGG UGG UAG CUC A UU-3′; RAD51 3′UTR siRNA, 5′-GUG CUG CAG CCU AAU GAG A dTdT-3′; TOPORS siRNA-1, 5′-CCC UGC UCC UUC AUA CGA A dTdT-3′; TOPORS siRNA-2, 5′-GCA GUA AGG AGG CCA ACU A dTdT-3′; TOPORS 3′UTR siRNA, 5′-CCA UAC GGU AUU GAC AUA U AdTdT-3′; ATM siRNA, 5′-UUC UCU UGC AAU CUC AUC AGG ACG C dTdT-3′; ATR siRNA, 5′-AAG ACG GUG UGC UCA UGC GGC dTdT-3′; and negative control siRNA (Bioneer, Republic of Korea), 5′-CCUACGCCACCAAUUUCGUdTdT-3′. For generation of stable TOPORS-depleted cell lines, oligonucleotides encoding the target sequences for TOPORS were annealed and inserted into psilencer2.1-U6-neo vector (Thermo Fisher Scientific). TOPORS shRNA-1: sense, 5′-GATCCAACCCTGCTCCTTCATACGTTCAAGAGAAATTCGTATGAAGGAGCAGGGTTTTTTGGAAA-3′; antisense, 5′-AGCTTTTCCAAAAAACCCTGCTCCTTCATACGAATTTCTCTTGAACGTATGAAGGAGCAGGGTTG-3′; and TOPORS shRNA-2: sense, 5′-GATCCAAGCAGTAAGGAGGCCAACTTCAAGAGAAATAGTTGGCCTCCTTACTGCTTTTTTGGAAA-3′; antisense, 5′-AGCTTTTCCAAAAAAAGCAGTAAGGAGGCCAACTATTTCTCTTGAAGTTGGCCTCCTTACTGCTTG-3′. U2OS cells were transfected with pSilencer2.1-U6-neo control shRNA or pSilencer2.1-U6-neo TOPORS shRNA-1/2 using Lipofectamine 2000 (Invitrogen). During 2–3 weeks, transfected cells were selected in medium containing 400 μg/ml neomycin (Sigma-Aldrich), and then stably TOPORS knockdown clones were confirmed by western blot analysis of TOPORS.

### Plasmids and cloning

The full-length human RAD51 cDNA was obtained by PCR from human HeLa cDNA pools and cloned into a pcDNA3-3xHA vector. The wild-type (WT) RAD51 was amplified by PCR from human RAD51 cDNA and cloned into a pcDNA3-3xHA vector. The pEGFP-N3 plasmids encoding full-length and truncated TOPORS (437–573 and 491–574) were gifted by Dr Eric H. Rubin (36). A comprehensive list of all PCR primers used in this study can be found in Supplementary Table S2. The K57R/K70R and V264K mutants of RAD51 in pcDNA3-3xHA and T515A and T515E mutants of TOPORS in pEGFP-N3 were generated by site-directed mutagenesis (GENEART^®^ System, Invitrogen) using DNA oligos described in Supplementary Table S2. For measuring the effect of TOPORS on HR activity, the WT and T515A mutant of TOPORS were amplified from pEGFP-N3-TOPORS-WT and pEGFP-N3-TOPORS-T515A, respectively, and the PCR products were inserted into EcoRI and KpnI sites of pcDNA3.1 vector. All constructs were verified by sequencing. For *in vitro* pull-down and *in vitro* SUMOylation assays, the WT RAD51 or WT TOPORS was cloned into the pET 28a or pGEX4T-1 vector, respectively.

### Immunofluorescence analysis

Cells were seeded onto glass coverslips, treated with 5 Gy ionizing radiation (IR) or 2 mM HU, and incubated at 37°C for indicated time points. Cells were then fixed with 4% paraformaldehyde for 10 min and ice-cold 98% methanol for 5 min, followed by permeabilization with 0.3% Triton X-100 for 15 min at room temperature. The coverslips were then washed three times with phosphate-buffered saline (PBS), followed by freshly made blocking solution (5% bovine serum albumin in PBS) for 1 h at room temperature. Immunostaining with appropriated primary antibodies (Supplementary Table S1) was followed by further washing with PBS and then incubation with the appropriate Alexa Fluor 488-, Alexa Fluor 594- or Alexa Fluor 647-conjugated secondary antibodies. The coverslips were mounted in mounting solution with DAPI (Vectashield, USA). Fluorescence images were taken under a confocal microscope (Zeiss LSM 900; Carl Zeiss, Germany) and analyzed with Zeiss AIM Image software (Carl Zeiss). For foci quantification experiments, cells with >5 foci were counted for positive cells and then percentage was calculated among at least 100 cells. The error bars represent standard deviation (SD) in three independent experiments.

### Immunoblot and immunoprecipitation analysis

Cells were lysed in RIPA buffer [50 mM Tris–HCl (pH 7.5), 150 mM NaCl, 1% Nonidet P-40 (NP40), 0.5% sodium deoxycholate, 0.1% SDS, 1 mM DTT] containing protease inhibitors (Roche). Proteins were separated by SDS-PAGE and transferred onto a polyvinylidene fluoride membrane (PALL Life Sciences). The membranes were blocked for 1 h with TBST [10 mM Tris–HCl (pH 7.4), 150 mM NaCl, 0.1% Tween 20] containing 5% nonfat milk and then incubated with appropriate primary antibodies at 4°C overnight, followed by peroxidase-conjugated secondary antibodies for 1 h at room temperature. The bands were visualized by an ECL chemiluminescent detection system (iNtRON Biotechnology). For immunoprecipitation of protein complexes, cell extracts were precleared with A-Sepharose beads (17-0780-01, GE Healthcare, USA) and incubated with the appropriate antibodies. Immune complexes were then analyzed by immunoblotting. If DNase I was used, the lysates were treated with 100 μg/ml DNase I (Invitrogen) for 20 min at 37°C. All antibodies used are listed in Supplementary Table S1.

### *In vivo* SUMOylation assay

For the endogenous RAD51 SUMOylation assay, HeLa cells transfected with control siRNA, TOPORS siRNA, SUMO1/2/3 siRNA3 or RAD51 siRNA were treated with or without 5 Gy IR for 1 h. For the exogenous RAD51 SUMOylation assay, HEK293T cells transfected with 3xHA-RAD51 constructs (WT or K57R/K70R mutant), GFP-TOPORS constructs (WT, 437–574, 491–574, T515A or T515E), Ubc9 and 6xHis-SUMO1/2/3 were treated with or without 5 Gy IR for 1 h or 2 mM HU for 3 h. Cells were lysed in lysis buffer including 50 mM Tris–HCl (pH 7.4), 150 mM NaCl, 1% NP40, 2 mM EDTA, 2 mM EGTA, 20 mM *N*-ethylmaleimide (NEM) and 0.1% SDS with protease inhibitors at 4°C for 15 min. After centrifugation at 13 200 rpm at 4°C for 30 min, lysates were precleared with protein G-Sepharose beads (GE Healthcare) at 4°C for 2–3 h and centrifuged at 13 000 rpm for 10 min. The precleared lysates were then incubated with anti-RAD51 polyclonal antibody (sc-8349, Santa Cruz, USA; ab63801, Abcam, USA), anti-His polyclonal antibody (sc-804, Santa Cruz) or anti-HA monoclonal antibody (ab18181, Abcam, USA) at 4°C overnight. Fresh G-Sepharose beads were added and kept at room temperature for 3 h. The beads were washed three times in lysis buffer, 2× SDS-PAGE loading buffer was then added to the beads and they were denatured by heating at 95°C for 5 min. The immunocomplexes were loaded on SDS-PAGE followed by western blotting analysis with anti-SUMO1 polyclonal antibody (ab32058, Abcam), anti-RAD51 monoclonal antibody (05-530, Millipore), anti-His monoclonal antibody (sc-8036, Santa Cruz; ab18184 Abcam) or anti-HA polyclonal antibody (sc-805, Santa Cruz).

### *In vitro* SUMOylation assay

*In vitro* SUMOylation was carried out using SUMOylation kit (BML-UW8955-0001, Enzo Life Sciences, USA) according to the manufacturer’s instructions. Briefly, 6xHis-RAD51 and GST-TOPORS were purified by a bacterial expression system. The SUMOylation reaction was performed in a 20 μl total volume with SUMO E1/E2 mix, SUMO1/2/3, 400 ng of purified GST-TOPORS and 200 nM purified 6xHis-RAD51. Reaction buffer and Mg-ATP were then added and incubated at 37°C for 1 h. The reaction was stopped by addition of 2× SDS-PAGE gel loading buffer followed by heating to 95°C for 5 min. The SUMOylation of RAD51 was showed by immunoblotting.

### *In vivo* ubiquitination assay

For the *in vivo* ubiquitination assay, control and TOPORS siRNA-transfected HeLa cells were treated with 5 Gy of IR for 1 h. Ten micromolar MG132 (Sigma-Aldrich) was added as indicated 4 h before sample collection. Cells were incubated in reaction mixture containing 1% NP40, 150 mM NaCl, 50 mM Tris–HCl (pH 8.0), 10 mM NaF, 1 mM NaVO_4_, 5 mM EDTA, 1 mM EGTA, 1 mM DTT with 20 mM NEM and protease inhibitor at 4°C for 15 min. The cell extracts were subjected to immunoprecipitation and immunoblotting.

### *In vitro* binding assay

For the *in vitro* binding assay of RAD51 and TOPORS, bacterially purified 6xHis-RAD51 or 6xHis alone was immobilized onto Ni-NTA Agarose (Macherey-Nagel) for 4 h at room temperature. The beads were washed two times with TEN100 buffer [20 mM Tris (pH 7.4), 0.1 mM EDTA and 100 mM NaCl] and incubated with 3 μg purified bacterial TOPORS at 4°C overnight. The Ni beads were washed at least three more times with 10 bed volumes of TEN100 buffer, and the bound protein was separated by SDS-PAGE and analyzed by western blotting using anti-RAD51 or anti-TOPORS antibodies.

### Chromosomal aberration analysis

Control and TOPORS-depleted HeLa cells or RAD51-depleted HeLa cells reconstituted with either 3xHA-RAD51-WT or 3xHA-RAD51-K57R/K70R were treated with or without 2 Gy IR. Twenty-four hours after IR, 100 ng/ml colcemid (Sigma-Aldrich) was added to arrest the cells in metaphase. One hour after treatment, cells were harvested, gently resuspended in 40% of culture media for 10 min at 37°C and then fixed in methanol–acetic acid (3:1). After removal of supernatant, pellets were resuspended in fixative solution, dropped onto a glass slide and air-dried overnight. The slide was mounted in mounting medium with DAPI (Vectashield). The metaphase images were captured using a confocal microscope (Zeiss LSM 900; Carl Zeiss), and the number of breaks and gaps was analyzed with image software ZEN (Carl Zeiss). At least 50 chromosomes were analyzed, and representative images were shown.

### Clonogenic survival assay

After treatment with IR, HU, mitomycin C (Sigma-Aldrich) or camptothecin (Sigma-Aldrich), 5 × 10^2^ cells were immediately seeded onto a 60-mm dish in triplicate and grown for 2–3 weeks at 37°C in a CO_2_ incubator to allow colony formation. Colonies were stained with 2% methylene blue in 50% ethanol and number of colonies was counted. The percentage of clonogenic survival was calculated as the ratio of the plating efficiency of treated cells compared to untreated cells. Results of clonogenic survival were presented as the mean value ± SD for three independent experiments.

### Comet assay

Neutral single-cell agarose gel electrophoresis was performed for measurement of repair activity of DSBs. Cells were treated with 5 Gy of IR or 2 mM HU, followed by incubation in culture medium at 37°C for the indicated times. TREVIGEN comet assay kit (TREVIGEN Instructions, USA) was utilized to detect changes in repair activity. The stained slides with SYBR Green (Lonza, USA) were analyzed using a fluorescence microscope (Nikon) at 400× magnification. The average comet tail moment was scored for 40–50 cells/slide using a computerized image analysis system (Komet 5.5; Andor Technology, Nottingham, UK).

### HR assay using DR-GFP cells

HR was measured using DR-GFP cells as described previously (37). Briefly, DR-GFP U2OS cells were transfected with control siRNA, TOPORS siRNA, RAD51 siRNA, or TOPORS and RAD51 siRNAs using Lipofectamine RNAiMax (Invitrogen). For the rescue experiment, TOPORS-depleted DR-GFP U2OS cells were transfected with TOPORS-WT or TOPORS-T515A. At 12 h after transfection, cells were transfected with pCBA-I-SceI plasmid using the Turbofect transfection reagent (Thermo Fisher Scientific). After 48 h, cells were harvested and analyzed for GFP expression by flow cytometry (FACSCalibur, BD Biosciences). The data were analyzed using CellQuest Pro Software (BD Biosciences). For recovery of HR repair by reintroducing untagged RAD51-WT or RAD51-K57R/K70R, DR-GFP U2OS cells were transfected with control or RAD51 3′UTR siRNAs. At 12 h after transfection, RAD51-WT, RAD51-K57/70R and pCBA-I-SceI plasmids were transfected again into DR-GFP U2OS cells. GFP-positive cells were counted 48 h after transfection using flow cytometry (FACSCalibur). The data were analyzed by CellQuest Pro Software (BD Biosciences).

### NHEJ assay using EJ5-GFP cells

NHEJ was measured in HeLa-EJ5 cells as described previously (38). Briefly, EJ5-GFP HeLa cells were transfected with control siRNA or two different TOPORS siRNAs using Lipofectamine RNAiMax. At 12 h, cells were transfected with pCBA-I-SceI plasmid using the Turbofect transfection reagent. After 36 h, the NHEJ activity was measured by flow cytometry (BD FACSCalibur, USA). For each analysis, 10 000 cells were processed and experiments were repeated three independent times.

### *In situ* proximity ligation assay

HeLa cells were either mock treated or treated with 5 Gy of IR, and were fixed at 2 h in 4% formaldehyde for 15 min and 100% formaldehyde for 5 min, followed by permeabilization with 0.5% Triton X-100 for 15 min at room temperature. *In situ* proximity ligation assay (PLA) was performed using Duolink PLA technology (Sigma-Aldrich) according to the manufacturer’s instructions. Briefly, coverslip was blocked in Duolink blocking buffer for 1 h at room temperature and then incubated with the two primary antibodies. The primary antibodies used were as follows: rabbit anti-RAD51 (ab63801, Abcam, 1:100) and mouse monoclonal anti-TOPORS (H00010210-M01, Abnova, 1:100). The negative control used only one primary antibody. The coverslips were washed twice in PBS for 5 min; anti-mouse PLUS and anti-rabbit MINUS PLA probes (DU920004 and DUO92002, respectively, Sigma-Aldrich) were coupled to the primary antibodies for 1 h at 37°C. Next, amplification was performed using the ‘Duolink *In Situ* Detection Reagents Red’ (DUO92001, DUO92005, Sigma-Aldrich). Finally, coverslips were mounted using Vectashield mounting media (Vector Laboratories) containing DAPI and imaged on a Carl Zeiss LSM 900 confocal microscope.

### CGH chip assay

Genomic DNA of TOPORS^+/+^ and TOPORS^−/−^ MEF cells was isolated using AccuPrep^®^ Genomic DNA Extraction Kit (Bioneer) according to the manufacturer’s instructions. Array comparative genomic hybridization (CGH) analysis was performed using the Agilent’s Mouse CGH 1x1M Array (Agilent Technologies). Mouse genomic DNA (0.1 μg) from TOPORS^−/−^ MEF cells and reference DNA samples from TOPORS^+/+^ MEF cells were labeled with Cy3 and Cy5, respectively, and cohybridized at 65°C for 24 h. The hybridized array was scanned using Agilent’s DNA microarray scanner. The log_2_ ratio values of the probe signal intensities were calculated and plotted versus genomic position using Feature Extraction Software (Agilent Technologies).

### Statistical analysis

All data were analyzed with GraphPad Prism software and Microsoft Excel. Differences between two independent groups were tested with two-tailed paired Student’s *t*-test. For the nonparametric statistical test, a Mann–Whitney test was used. *P*-value of <0.01 was considered statistically significant and *P*-values were indicated by asterisks as follows: **P < 0.01 and ns = nonsignificant. Error bars represent SD of three independent experiments. All experiments were performed in triplicate, and repeated at least three times.

## RESULTS

### TOPORS interacts with RAD51 and accumulates at sites of DNA damage

**Figure 1.**
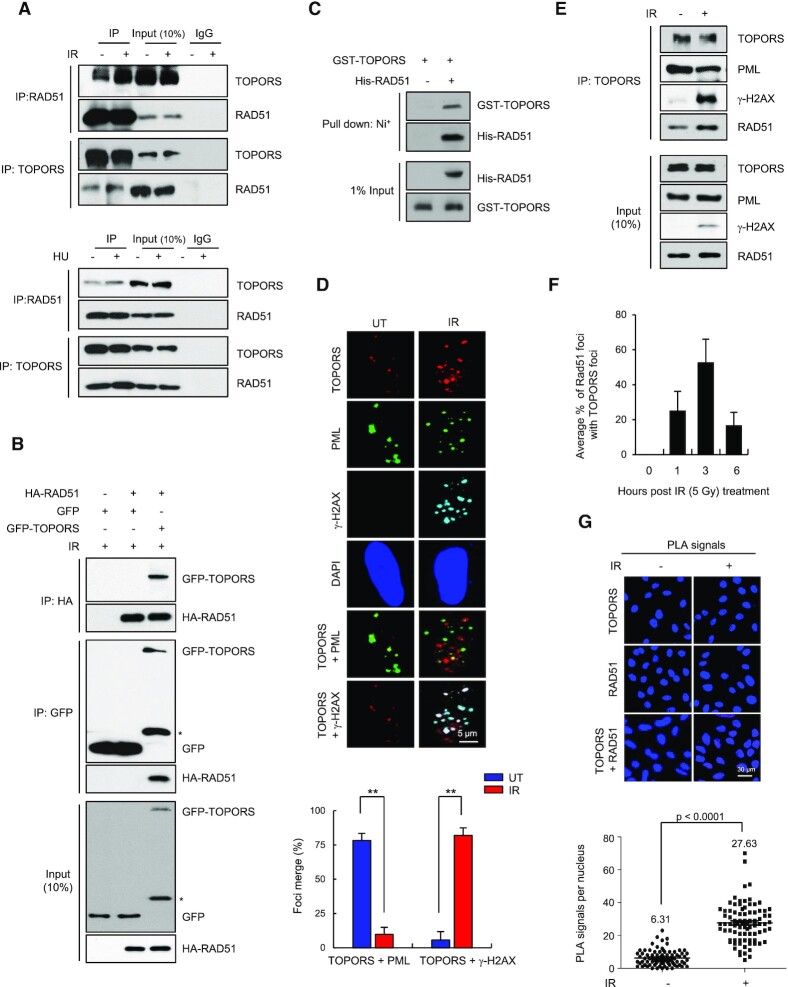
TOPORS interacts with RAD51. **(A)** HeLa cells, with or without 5 Gy of IR or 2 mM HU treatment, were lysed and subjected to immunoprecipitation followed by immunoblotting as indicated. **(B)** HEK293T cells transfected with HA-RAD51 along with or without GFP-TOPORS were treated with 5 Gy of IR and subjected to immunoprecipitation and immunoblots as indicated. Asterisk indicates nonspecific band. **(C)***In vitro* pull-down assay carried out using immobilized control His or His-RAD51 fusion proteins on Ni^+^-NTA Agarose followed by recombinant GST-TOPORS protein. GST pull-downs were immunoblotted with antibodies as indicated. **(D)** HeLa cells, treated with or without 5 Gy of IR, were stained with indicated antibodies. Colocalization of TOPORS (red) and PML (green) is visible as a yellow merged signal, and colocalization of TOPORS (red) and γ-H2AX (cyan) is visible as a white merged signal. Nuclei were stained with DAPI. Scale bar: 5 μm. The percent colocalization between TOPORS and PML and between TOPORS and γ-H2AX is shown. Quantification of merged foci as a percentage of TOPORS–PML or TOPORS–γ-H2AX foci from total TOPORS foci. At least 50 cells from three independent slides were analyzed. The results are shown as mean ± SD (*n* = 3), ^∗∗^*P* < 0.01. **(E)** Whole cell lysates of HeLa cells, with or without 5 Gy of IR treatment, immunoprecipitated using an anti-TOPORS antibody followed by immunoblotting using indicated antibodies. **(F)** Percent colocalization of RAD51 and TOPORS foci at the indicated times following IR treatment. Data are presented as mean ± SD (*n* = 3). **(G)** A PLA probe was used to detect colocalization of endogenous RAD51–TOPORS. DNA were counterstained with DAPI. The negative controls were obtained by omitting one of the primary antibodies. Representative images and a scatterplot of the PLA signal per nucleus are shown. Scale bar: 30 μm. Data are presented as mean ± SD (*n* =3). *P*-values between indicated samples were calculated using a Mann–Whitney test.

In an attempt to identify new regulators of RAD51, we exploited a yeast two-hybrid screen using human RAD51 as bait and identified several binding proteins (Supplementary Figure S1A and B). Among them, TOPORS was particularly notable because it has a SUMO E3 ligase activity (39–42), while HR repair is known to be regulated by SUMOylation (43). Further, TOPORS deficiency is known to cause a high rate of aneuploidy and an increased rate of malignancy (36). In order to investigate the interaction further, we confirmed the interaction between endogenous RAD51 and TOPORS in human cells using co-immunoprecipitation assays (Figure 1A). We repeatedly noted that the interaction was enhanced by IR and HU treatment (Figure 1A). Additionally, we found that ectopically expressed HA-tagged RAD51 and GFP-tagged TOPORS coprecipitated (Figure 1B), further suggesting that these two proteins interact in cells. Treatment of lysates with DNase did not alter the interaction between RAD51 and TOPORS, suggesting that the interaction is not mediated by DNA (Supplementary Figure S1C–E). These two proteins are able to interact directly, as bacterially purified His-RAD51 pulls down GST-TOPORS *in vitro* (Figure 1C).

Prompted by the results, we investigated whether TOPORS also accumulates at the DSB sites. Previous studies showed that TOPORS accumulates in several nuclear aggregates, colocalizing with promyelocytic leukemia protein (PML) nuclear bodies in undamaged cells, which we observed consistently (Figure 1D) (44). Interestingly, treatment of cells with IR led to extensive overlap between the endogenous TOPORS foci and those of the DSB marker γ-H2AX (Figure 1D). Coincidentally, the overlap between TOPORS and the PML bodies was lost, suggesting that TOPORS migrates away from the PML body upon DNA damage. Co-immunoprecipitation analyses confirmed the physical association between TOPORS and γ-H2AX following irradiation (Figure 1E). Further, we observed that TOPORS closely colocalized with RAD51 after exposure to IR (Figure 1F and Supplementary Figure S1F). Moreover, a PLA-based approach indicated a significant increase in the number of RAD51/TOPORS PLA foci in IR-treated HeLa cells as compared with untreated cells (Figure 1G). Taken together, these data suggest that TOPORS is a RAD51-interacting protein that localizes to DSBs upon exposure to DNA damaging agents.

### TOPORS–RAD51 interaction is enhanced by ATM-induced phosphorylation of TOPORS

**Figure 2.**
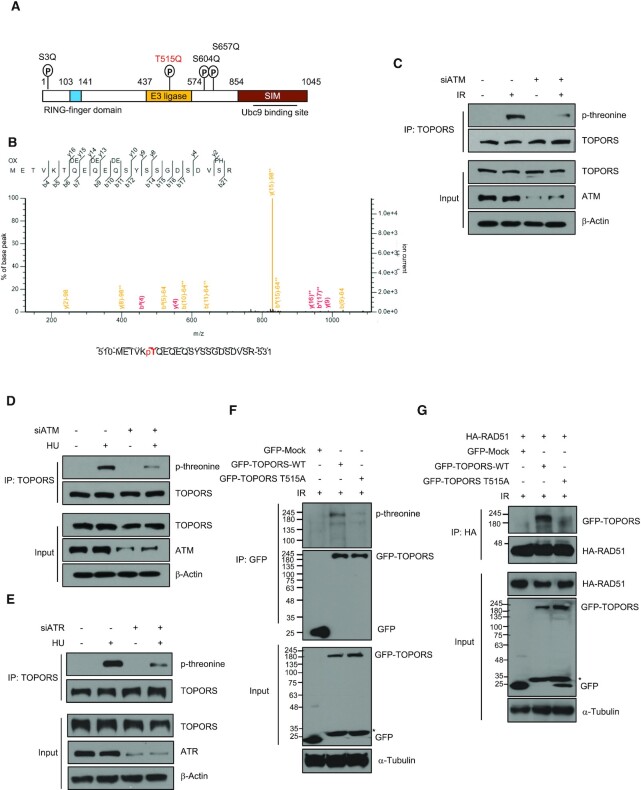
TOPORS is phosphorylated at Thr515 in response to IR. **(A)** Schematic diagrams of the TOPORS protein domains with putative ATM phosphorylation sites indicated. **(B)** Determination of IR-induced phosphorylation sites in TOPORS by mass spectrometry. A peptide containing Thr515 phosphorylation is shown. HeLa cells transfected with control siRNA or ATM siRNA were treated with or without 5 Gy of IR (**C**) or 2 mM HU (**D**). Whole cell lysates were then subjected to immunoprecipitation using an anti-TOPORS antibody followed by immunoblotting using indicated antibodies. **(E)** HeLa cells transfected with control siRNA or ATR siRNA were treated with or without 2 mM HU. Whole cell lysates were then subjected to immunoprecipitation using an anti-TOPORS antibody followed by immunoblotting using indicated antibodies. **(F)** HEK293T cells transfected with control GFP vector, GFP-TOPORS-WT or GFP-TOPORS-T515A were treated with IR (5 Gy). Cell lysates were immunoprecipitated with anti-GFP antibody and subjected to immunoblot analysis with anti-phospho-threonine antibody. Asterisk indicates degradation products of GFP-TOPORS. **(G)** HA-RAD51-expressing HEK293T cells transfected with control GFP vector, GFP-TOPORS-WT or GFP-TOPORS-T515A were treated with 5 Gy of IR and subjected to immunoprecipitation and immunoblots as indicated. Asterisk indicates degradation products of GFP-TOPORS.

Factors involved in DDR are often phosphorylated on SQ/TQ motifs by kinases ATM or ATR (45). We noted that these motifs are found in the TOPORS sequence: serine 3 (Ser3), threonine 515 (Thr515), serine 604 (Ser604) and serine 657 (Ser657) (Figure 2A). Interestingly, mass spectrometry analysis found that Thr515, but not Ser3, Ser604 and Ser657, was phosphorylated after IR treatment (Figure 2B). Using an antibody that recognizes phosphorylated threonine, we found that threonine was phosphorylated in response to IR and HU, and the levels of phosphorylated threonine decreased significantly after ATM knockdown (Figure 2C and D). In addition, the levels of HU-induced phosphorylated threonine decreased after ATR knockdown (Figure 2E). To confirm that Thr515 is the major phosphorylated residue of TOPORS after DNA damage, it was replaced with alanine (T515A) by mutagenesis. This resulted in a marked decrease in the phosphorylation compared to the WT TOPORS (Figure 2F). Next, we sought to test whether the Thr515 phosphorylation affects the interaction between TOPORS and RAD51. We found that the presence of the T515A mutation impaired interactions with RAD51 (Figure 2G), while phosphomimetic mutant T515E readily bound to RAD51 (Supplementary Figure S2). These results altogether suggest that TOPORS is phosphorylated by ATM and ATR, and that the phosphorylation regulates the TOPORS–RAD51 interaction.

### TOPORS is required for RAD51 chromatin recruitment, HR repair and genomic integrity

**Figure 3.**
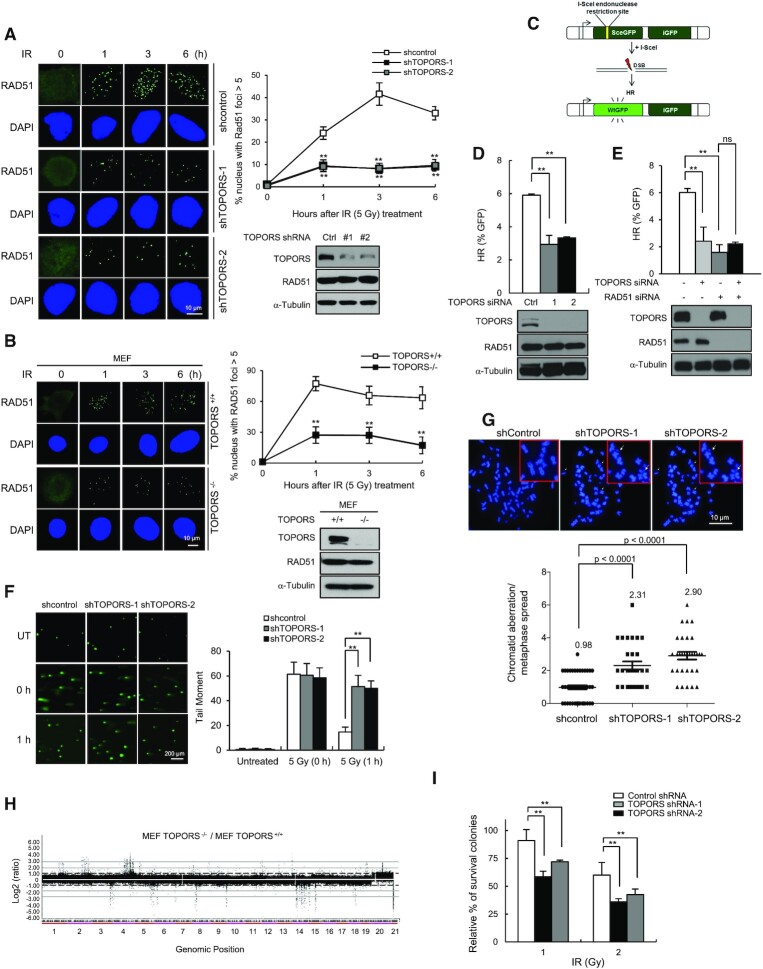
TOPORS plays an important role in DSB repair. Control and TOPORS-depleted U2OS cells (**A**) or control and TOPORS^−/−^ MEF cells (**B**) were exposed to 5 Gy of IR and fixed at the indicated time points. Immunostaining experiments were performed using an anti-RAD51 antibody. DNA was counterstained with DAPI. Scale bar: 10 μm. Percentage of cell populations that shows >5 foci for RAD51 is shown. The results are shown as mean ± SD (*n* = 3), ^∗∗^*P* < 0.01. **(C)** A schematic of the DR-GFP reporter system used to measure rates of HR. **(D)** HR efficiency as measured by FACS in control and TOPORS-depleted U2OS/DR-GFP cells. The results are shown as mean ± SD (*n* = 3), ^∗∗^*P* < 0.01. **(E)** HR efficiency of U2OS/DR-GFP cells transfected with the indicated siRNA combinations. The results are shown as mean ± SD (*n* = 3), ^∗∗^*P* < 0.01, ns = not significant. **(F)** IR-induced DNA damage as measured through a neutral comet assay in control and TOPORS-depleted U2OS cells. The results are shown as mean ± SD (*n* = 3), ^∗∗^*P* < 0.01. **(G)** The number of chromosome aberrations as measured by metaphase chromosome spreads of control and TOPORS-depleted U2OS cells treated with 2 Gy of IR. Representative images and quantification of aberrations are shown. The results are shown as mean ± SD (*n* = 3). *P*-values between the indicated samples were calculated using a Mann–Whitney test. **(H)** Array CGH profiles of genomic DNA derived from TOPORS^+/+^ and TOPORS^−/−^ MEFs. Genomic positions that fall above or below the dotted line indicate amplifications or deletions of regions of genome, respectively. **(I)** Colony forming ability of control and TOPORS-depleted U2OS cells treated with the indicated doses of IR. The results are shown as mean ± SD (*n* = 3), ^∗∗^*P* < 0.01.

To determine the functional consequences of the RAD51–TOPORS interaction, we investigated the effect of TOPORS on IR-induced RAD51 foci formation. We generated two U2OS cell lines that stably integrated TOPORS-targeting shRNAs (shTOPORS-1 and shTOPORS-2), and confirmed the protein levels of TOPORS were decreased (Figure 3A). In cells expressing control shRNA, IR treatment led to the formation of discrete RAD51 nuclear foci, as expected. However, in TOPORS-depleted cells, significantly fewer IR-induced RAD51 foci were visible (Figure 3A). Similar results were obtained in embryonic fibroblasts from TOPORS knockout mice (TOPORS^−/−^ MEFs) (Figure 3B). On the other hand, TOPORS knockdown did not affect formation of the IR-induced 53BP1 foci (Supplementary Figure S3A). It is to be noted that the overall RAD51 expression levels remain unchanged upon TOPORS depletion. The IR-induced RAD51 foci formation was rescued through the expression of exogenous TOPORS in cells depleted of endogenous TOPORS by an siRNA targeting the 3′UTR (Supplementary Figure S3B), confirming a critical role for TOPORS in IR-induced RAD51 nuclear foci formation.

To test whether decreased RAD51 foci formation leads to HR repair defects, we used the HR reporter cell line in which gene homologous conversion rate is determined by recovery of WT GFP molecules from two different GFP mutants oriented in direct repeats (37) (Figure 3C). Upon DSB induction (I-Sce1), there was ∼2-fold reduction in the GFP+ in TOPORS-depleted cells, consistently suggesting the HR repair impairment upon TOPORS depletion (Figure 3D and Supplementary Table S3). There was no effect on the NHEJ repair efficiency by TOPORS depletion (Supplementary Figure S3C and D), suggesting that TOPORS specifically affects the HR repair. No additional decrease in HR was observed when TOPORS and RAD51 were depleted simultaneously (Figure 3E and Supplementary Table S3), possibly suggesting that TOPORS regulates HR repair mainly through regulating RAD51.

We also performed the neutral comet assays, and found that either knockdown or knockout of TOPORS caused a delay in disappearance of comet tails after IR treatment (Figure 3F and Supplementary Figure S3E), and one interpretation could be that TOPORS is required for repair of DSBs. Introduction of GFP-TOPORS complemented the DSB repair defects in TOPORS-depleted cells (Supplementary Figure S3F). Furthermore, we detected significantly higher frequencies of chromosomal aberrations in TOPORS knockdown cells compared with control cells (Figure 3G). Using array CGH, we showed that TOPORS knockout MEF cells had a significantly higher number of chromosomal abnormalities, including clonal amplifications (dot above +1.0) and deletions (dot below −1.0), that were widely distributed throughout the genome (Figure 3H), indicating that TOPORS is critical for the maintenance of chromosome stability. Consistently, a knockdown of TOPORS led to reduced cell viability upon IR, mitomycin C and camptothecin treatment (Figure 3I and Supplementary Figure S3G and H). Collectively, these results indicate that TOPORS plays a critical function in the RAD51-mediated HR and overall chromosomal integrity, likely by promoting the recruitment of RAD51 to sites of DNA damage.

### TOPORS induces SUMOylation of RAD51

**Figure 4.**
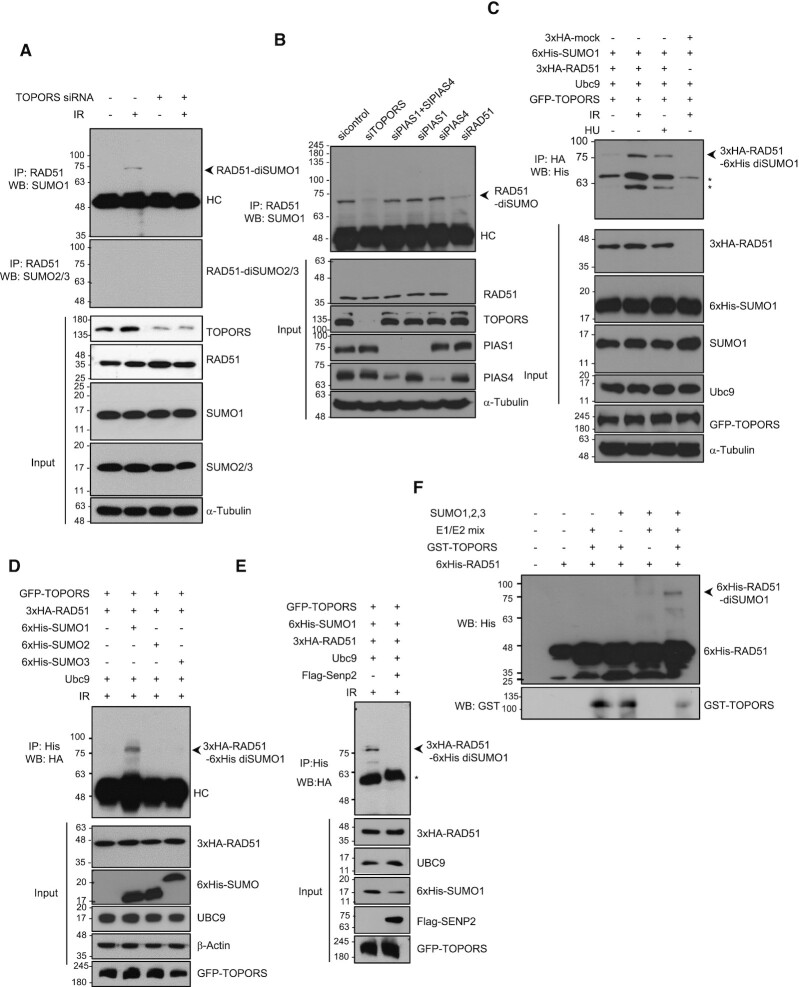
RAD51 is SUMOylated both *in vitro* and *in vivo*. **(A)** Control and TOPORS-depleted HeLa cells were treated with or without 5 Gy of IR. Immunoprecipitations using the anti-RAD51 antibody were performed and the following immunoblot analyses were done using anti-SUMO1 and anti-SUMO2/3 antibodies. HC indicates heavy chain. **(B)** HeLa cells transfected with control, TOPORS, PIAS1 and PIAS4, PIAS1, PIAS4 or RAD51 siRNA were irradiated with 5 Gy of IR, immunoprecipitated with anti-RAD51 antibody and subjected to immunoblot analysis with anti-SUMO1 antibody. HC indicates heavy chain. **(C)** HEK293T cells were cotransfected with HA-RAD51, GFP-TOPORS, His-SUMO1 and Ubc9, treated with IR (5 Gy) or HU (2 mM), and subjected to immunoprecipitation and immunoblotting as indicated. Asterisks indicate nonspecific bands. **(D)** HEK293T cells were transfected with His-SUMO1, His-SUMO2 or His-SUMO3 along with the indicated plasmids, exposed to 5 Gy of IR and subjected to immunoprecipitation followed by immunoblotting as indicated. HC indicates heavy chain. **(E)** HEK293T cells transfected with control or Flag-Senp2 along with the indicated plasmids were exposed to 5 Gy of IR and subjected to immunoprecipitation and immunoblotting as indicated. Asterisk indicates nonspecific band. **(F)** His-tagged RAD51 proteins incubated with purified GST-tagged TOPORS, recombinant SUMO1/2/3 and/or SUMO E1/E2 as indicated and analyzed by immunoblotting using anti-His and anti-GST antibodies.

Given that TOPORS has both SUMO1 and ubiquitin E3 ligase activities (39,41,46,47), we hypothesized that TOPORS controls HR repair by modifying RAD51 through one of these two activities. We noted that knockdown of TOPORS did not seem to majorly affect the polyubiquitination of endogenous RAD51 in response to IR (Supplementary Figure S4A). However, we surprisingly found that an anti-SUMO1 antibody, but not anti-SUMO2/3 antibody, detected a molecular weight of ∼70 kDa, a possible size of di-SUMOylated RAD51, in cells treated with IR; a theoretical molecular weight of SUMO1 molecule is 11 kDa, but the SUMO modification added to a protein on SDS-PAGE is typically in the range of 15–17 kDa (48). We provide the expected molecular weight chart of RAD51 SUMOylation on SDS-PAGE in Supplementary Figure S4B. Importantly, this band was undetectable in TOPORS knockdown cells (Figure 4A). The ∼70 kDa band was dependent on SUMO1 conjugation, as it was absent in cells transfected with SUMO1 siRNA, but not with SUMO2 or SUMO3 siRNAs (Supplementary Figure S4C). This result agrees with previous reports suggesting that TOPORS is a SUMO1 E3 ligase (40,42). Two E3 SUMOylating enzymes known to participate in DNA damage response known are PIAS1 and PIAS4 (43). We found that knockdown of PIAS1, PIAS4 or both genes simultaneously did not affect the RAD51 SUMOylation (Figure 4B), with a caveat that incompleteness of PIAS4 knockdown does not allow completely ruling out its possible role in RAD51 SUMOylation. These data argue that TOPORS is a major SUMO E3 ligase that is responsible for RAD51 SUMOylation.

To further verify that this protein was a SUMO1 conjugated form of RAD51, we transiently transfected GFP-TOPORS, 3xHA-RAD51 and E2 conjugating enzyme UBC9 together with 6xHis-SUMO1 into HEK293T cells and treated with IR or HU. The resulting ∼80 kDa band (3xHA tagging of RAD51 and 6xHis tagging of SUMO account for the slight increase in the size in comparison to the bands in Figure 4A and B), corresponding to di-SUMO-conjugated 3xHA-RAD51, was detected at higher levels upon exposure to IR or HU (Figure 4C). This band was present in transfectants expressing 6xHis-SUMO1, but not in those expressing 6xHis-SUMO2 and 6xHis-SUMO3 (Figure 4D). Loss of this band in the presence of SUMO protease 2 (SENP2) overexpression further supported that it is the SUMOylation modification (Figure 4E). The band is also lost upon siRAD51 transfection (Figure 4B, last lane, and Supplementary Figure S4D), confirming that the modification occurred on the RAD51 protein. To determine whether TOPORS directly SUMOylates RAD51, we performed *in vitro* SUMOylation assay using defined components. Consistent with the above assays using cell lysates, a band of ∼70 kDa was observed that is dependent on SUMO1 (but not SUMO2 or SUMO3), the SUMO enzyme mix and TOPORS (Figure 4F). There is a possibility of mono-SUMO species, but this was not as evident as di-SUMOylation.

### SUMOylation enhances RAD51 functions

**Figure 5.**
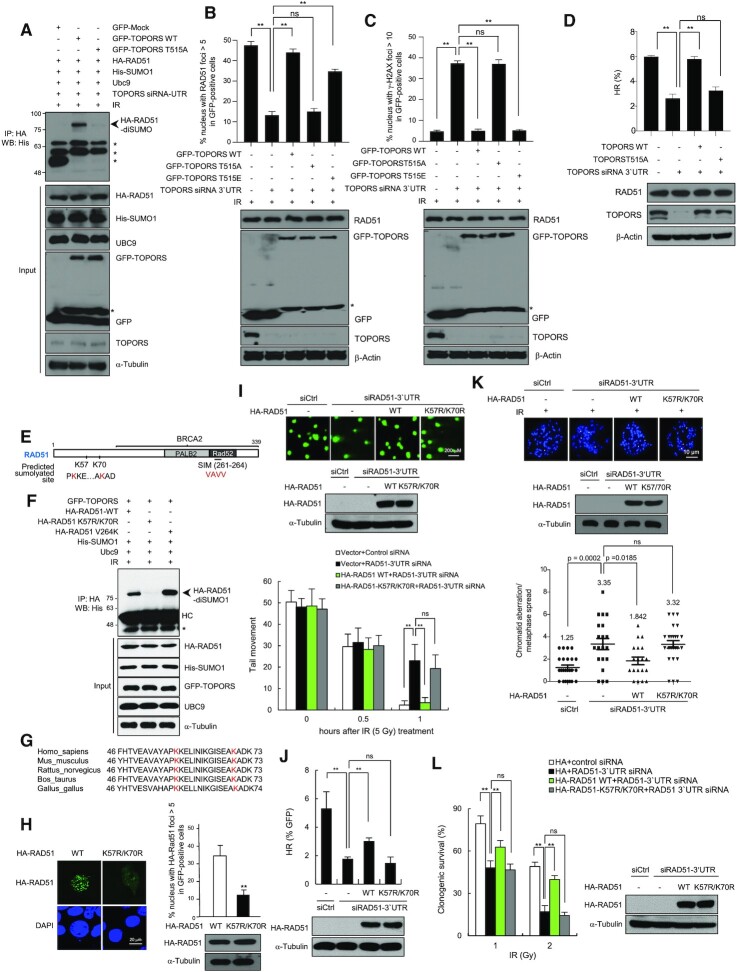
RAD51 SUMOylation is required for HR-mediated DSB repair. **(A)** TOPORS knockdown HEK293T cells were transfected with GFP-TOPORS-WT or GFP-TOPORS (T515A) along with the indicated plasmids, exposed to 5 Gy of IR, and immunoprecipitated and immunoblotted as indicated. Asterisks indicate nonspecific bands. **(B)** TOPORS knockdown HeLa cells reconstituted with GFP-TOPORS-WT or GFP-TOPORS (T515A or T515E) were treated with 5 Gy of IR, fixed at 3 h and immunostained using an anti-RAD51 antibody. The percentage of cell populations that shows >10 foci for RAD51 in GFP-positive cells is shown. The results are shown as mean ± SD (*n* = 3), ^∗∗^*P* < 0.01, ns = not significant. Asterisk indicates degradation products of GFP-TOPORS. **(C)** γ-H2AX foci of the same cells as described in (B). Cells were treated with 5 Gy of IR, fixed at 24 h and immunostained using an anti-γ-H2AX. The percentage of cell populations that shows >10 foci for γ-H2AX in GFP-positive cells is shown. The results are shown as mean ± SD (*n* = 3), ^∗∗^*P* < 0.01, ns = not significant. Asterisk indicates degradation products of GFP-TOPORS. **(D)** HR efficiency of TOPORS knockdown U2OS/DR-GFP cells reconstituted with TOPORS-WT or TOPORS (T515A). The results are shown as mean ± SD (*n* = 3), ^∗∗^*P* < 0.01, ns = not significant. **(E)** A schematic of the domains of the human RAD51 including two putative SUMOylation sites and the SUMO-interacting motif (SIM). **(F)** HEK293T cells transfected with HA-RAD51-WT or mutants together with indicated plasmids were exposed to 5 Gy of IR. Whole cell lysates were analyzed by immunoprecipitation followed by immunoblotting as indicated. Asterisk indicates nonspecific band and HC indicates heavy chain. **(G)** An amino acid sequence alignment of the predicted consensus SUMO site with K57 and K70 highlighted in red. **(H)** HeLa cells expressing HA-RAD51-WT or HA-RAD51-K57R/K70R were treated with 5 Gy of IR, fixed at 3 h and immunostained using an anti-HA antibody. Nuclei were stained with DAPI. Scale bar: 10 μm. The percentage of cell populations that shows >5 foci for RAD51 is shown. The results are shown as mean ± SD (*n* = 3), ^∗∗^*P* < 0.01. **(I)** Quantification of DNA damage through a neutral comet assay in control, RAD51 knockdown and RAD51 knockdown cells expressing indicated constructs after treatment with IR (5 Gy) at the indicated time points. Scale bar: 200 μm. The results are shown as mean ± SD (*n* = 3), ^∗∗^*P* < 0.01, ns = not significant. **(J)** The efficiency of HR repair, measured using the assay depicted in Figure 3C, in RAD51-depleted DR-GFP-U2OS cells reconstituted with the indicated constructs. The results are shown as mean ± SD (*n* = 3), ^∗∗^*P* < 0.01, ns = not significant. **(K)** RAD51 knockdown HeLa cells reconstituted with HA-RAD51-WT and HA-RAD51-K57R/K70R were treated with 2 Gy of IR. The number of chromosome aberrations was measured by metaphase chromosome spreads. The results are shown as mean ± SD (*n* = 3). *P*-values between the indicated samples were calculated using a Mann–Whitney test; ns = not significant. **(L)** Colony forming ability of the same cells as described in (J). Cells were treated with the indicated doses of IR. The results are shown as mean ± SD (*n* = 3), ^∗∗^*P* < 0.01, ns = not significant.

Given that the phosphorylation of TOPORS at Thr515 facilitates its interaction with RAD51 (Figure 2E), we hypothesized that TOPORS phosphorylation affects RAD51 SUMOylation. To test this, we reconstituted TOPORS-depleted cells with GFP-TOPORS-WT, GFP-TOPORS-T515A or GFP-TOPORS-T515E constructs. We found that GFP-TOPORS-WT, but not GFP-TOPORS-T515A, restored the RAD51 SUMOylation upon IR treatment (Figure 5A), suggesting that phosphorylation of TOPORS Thr515 plays a vital role in regulating RAD51 SUMOylation. GFP-TOPORS-T515E also was able to restore the SUMOylation (Supplementary Figure S5), suggesting that it is the lack of phosphorylation that inhibits the SUMOylation, rather than lack of threonine causing other effects. We further investigated the biological significance of Thr515 phosphorylation of TOPORS during DSB repair. GFP-TOPORS-WT, GFP-TOPORS-T515A mutant or GFP-TOPORS-T515E mutant was reconstituted into cells in which endogenous TOPORS was knocked down. We found that TOPORS-T515A mutant, but not TOPORS-WT and TOPORS-T515E mutant, significantly compromised RAD51 foci formation upon IR treatment (Figure 5B). Consistently, TOPORS-T515A mutant, but not TOPORS-WT and TOPORS-T515E mutant, failed to rescue the DSB repair in TOPORS-depleted cells (Figure 5C). Moreover, TOPORS-T515A mutant did not rescue the HR repair in TOPORS-depleted cells (Figure 5D).

Using the SUMOsp 2.0 (http://sumosp.biocuckoo.org/) program, we identified two consensus SUMO-targeting motifs in RAD51 at lysine 57 (K57) and lysine 70 (K70) (Figure 5E). SUMOylation of these sites in response to IR was assayed using a double (K57R/K70R) RAD51 mutant in HEK293T cells. Immunoblot analysis of HA pull-down samples revealed efficient SUMOylation of WT RAD51 but not of the K57R/K70R mutant (Figure 5F). It was previously reported that RAD51 interacts with SUMO1 noncovalently through the V264 residue within the SIM (35). Mutation of the V264 residue did not affect the SUMO modification (Figure 5E, third lane), consistent with a prediction that the SIM itself is not a modification site. The K57 and K70 residues in RAD51 are well conserved across species, including mouse, rat, cow, chicken and human (Figure 5G), which is further evidence of a key functional role for these residues.

To address the functional significance of RAD51 SUMOylation by TOPORS, we examined whether loss of SUMOylation at residues K57 and K70 affects the recruitment of RAD51 to DSB sites. Using HeLa cells expressing either 3xHA-RAD51-WT or the 3xHA-RAD51-K57R/K70R mutant, we investigated the formation of nuclear foci. HA-RAD51-WT efficiently formed foci after exposure to IR, as expected. In contrast, 3xHA-RAD51-K57R/K70R foci formation was severely compromised (Figure 5H), indicating that the SUMOylation status of RAD51 at these residues is important for its correct recruitment to DSB sites.

Given that RAD51 SUMOylation plays a significant role in the efficient accumulation of RAD51 at sites of DSBs, we asked whether the rate of DSB repair was affected. A neutral comet assay was used to measure DSBs following IR exposure in HeLa cells in which endogenous RAD51 was replaced with either 3xHA-RAD51-WT or 3xHA-RAD51-K57R/K70R. Cells lacking endogenous RAD51 were deficient in DSB repair. However, while the presence of 3xHA-RAD51-WT fully restored DSB repair at 1 h following IR treatment, much less repair was observed in the presence of 3xHA-RAD51-K57R/K70R (Figure 5I). Likewise, when RAD51-depleted DR-GFP U2OS cells were complemented with either 3xHA-RAD51-WT or 3xHA-RAD51-K57R/K70R, only HA-RAD51-WT rescued the impaired HR (Figure 5J).

To further corroborate these findings, we assessed chromatid aberrations in mitotic spreads at 24 h after IR exposure. IR treatment led to significantly higher numbers of chromatid aberrations in RAD51 knockdown cells compared to control cells (Figure 5K), as expected. The number of chromatid aberrations returned to normal when RAD51 knockdown cells were complemented with RAD51-WT, but not when complemented with RAD51-K57R/K70R. The physiological relevance of RAD51 SUMOylation was further explored through clonogenic survival assays performed using IR-exposed RAD51-depleted HeLa cells reconstituted with either 3xHA-RAD51-WT or 3xHA-RAD51-K57R/K70R. RAD51-WT restored cell survival to a level similar to control siRNA-transfected cells, whereas RAD51-K57R/K70R did not (Figure 5L). Together, these data indicate that SUMOylation of RAD51 at K57 and K70 enhances the normal functioning of RAD51 in HR.

### RAD51 SUMOylation promotes DSB repair at stalled replication forks

Prolonged fork stalling in response to HU treatment leads to fork collapse and replication-coupled DSBs, and RAD51 plays an important role in repairing the DSBs that arise during these types of replication stress (10,49). A role for SUMOylation in this process was evidenced by the observation that RAD51 SUMOylation was stimulated by HU (Figure 4C) and that this effect was greatly reduced in the presence of the K57R/K70R mutant (Supplementary Figure S6A). To evaluate DSB repair of stalled forks, we measured comet tail moments after release from a 24 h treatment with HU, which induces DSBs at stalled forks (10). We found that the number of HU-induced DSBs was significantly higher in cells depleted of endogenous RAD51 and that this effect could be rescued through the expression of exogenous RAD51-WT, but not RAD51-K57R/K70R (Supplementary Figure S6B). We then evaluated viability after prolonged HU treatment using a clonogenic survival assay and found that RAD51-WT but not RAD51-K57R/K70R corrected the HU sensitivity induced through depletion of endogenous RAD51 (Supplementary Figure S6C). Unrecovered HU-induced DNA breakage and cellular sensitivity to HU may result from impaired RAD51 recruitment to stalled replication forks. To test this, HU-induced foci of 3xHA-RAD51-WT and 3xHA-RAD51-K57R/K70R were measured. As expected (10), in cells exposed to HU for 24 h, HA-RAD51-WT formed foci normally (Supplementary Figure S6D). On the other hand, the recruitment of 3xHA-RAD51-K57R/K70R to stalled replication forks was severely impaired. Moreover, when TOPORS knockdown cells were exposed to HU, both RAD51 foci formation and cell survival were also compromised (Supplementary Figure S6E and F). These data suggest that K57/K70 SUMOylation may serve as a critical event for the correct functioning of RAD51 during the repair of DSBs at stalled replication forks.

### SUMOylation regulates the RAD51–BRCA2 interaction

**Figure 6.**
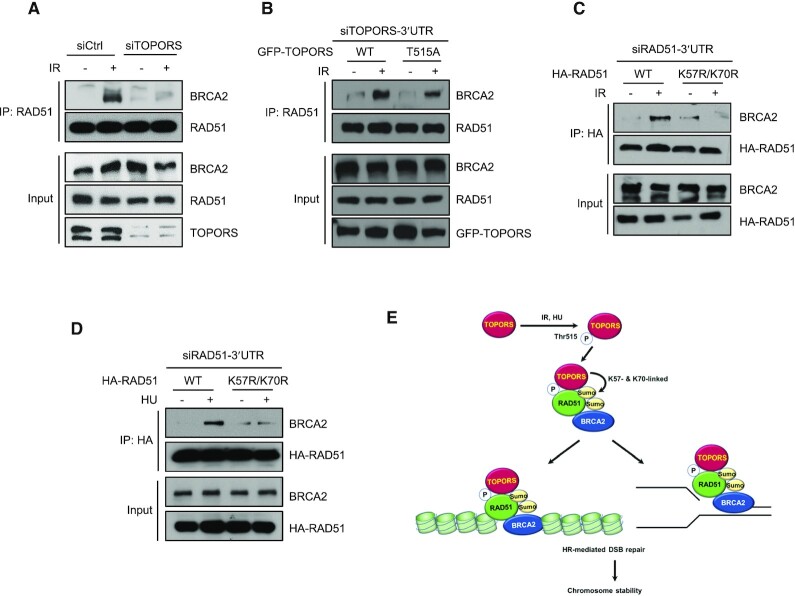
SUMOylation of RAD51 promotes its interaction with BRCA2. **(A)** Control and TOPORS-depleted HeLa cells were treated with or without 5 Gy of IR. Immunoprecipitations using an anti-RAD51 antibody were performed and the following immunoblot analyses were done using anti-BRCA2 or anti-RAD51 antibodies. **(B)** TOPORS knockdown HEK293T cells reconstituted with GFP-TOPORS-WT or GFP-TOPORS (T515A) were treated with or without IR (5 Gy), and subjected to immunoprecipitation and immunoblotting as indicated. RAD51 knockdown HeLa cells reconstituted with HA-RAD51-WT and HA-RAD51-K57R/K70R were treated with or without 5 Gy of IR **(C)** or 2 mM HU **(D)**, and subjected to immunoprecipitation and immunoblotting as indicated. **(E)** Schematic model representing the role of TOPORS in RAD51 function in DSBs and replication stress. See text for details.

Next, we wished to determine how TOPORS or SUMOylation of RAD51 regulates its functions, particularly its recruitment to DSBs, during HR. As BRCA2 binds directly to RAD51 and facilitates the loading of RAD51 onto ssDNA (2,12–15), we tested whether the binding between RAD51 and BRCA2 is affected by TOPORS. Interestingly, knockdown of TOPORS compromised the association between RAD51 and BRCA2 (Figure 6A and Supplementary Figure S7A). Next, we asked whether phosphorylation of TOPORS at Thr515 is involved in regulation of the interaction between RAD51 and BRCA2. To this end, cells expressing either GFP-TOPORS-WT or GFP-TOPORS-T515A were treated with TOPORS 3′UTR siRNA to deplete endogenous TOPORS, and then analyzed for interaction between RAD51 and BRCA2 following IR treatment. We found that reconstitution of TOPORS-depleted cells with GFP-TOPORS-WT restored the interaction between RAD51 and BRCA2, but the T515A mutant restored to a lesser degree (Figure 6B and Supplementary Figure S7B), suggesting that phosphorylation of TOPORS at Thr515 enhances the interaction between RAD51 and BRCA2. We then examined the effects of RAD51 SUMOylation on its interaction with BRCA2. We overexpressed 3xHA-RAD51-WT or 3xHA-RAD51-K57R/K70R in RAD51-depleted cells and then examined its interaction with BRCA2 following IR or HU treatment. As expected, there was a significant increase in the association of BRCA2 and 3xHA-RAD51-WT (Figure 6C and D, and Supplementary Figure S7C and D). On the other hand, there was markedly less BRCA2 detected in the presence of 3xHA-RAD51-K57R/K70R. These results highlight a critical role for SUMOylation of RAD51 at K57 and K70 in regulating the interaction between RAD51 and BRCA2, thereby providing molecular insight into how SUMOylation promotes recruitment of RAD51 to DNA lesions.

## DISCUSSION

Herein, we present a set of data demonstrating that SUMO modification of RAD51 is a crucial event during the HR repair of DSBs. Through an unbiased screen, we identified that TOPORS is a new interactor of RAD51. We showed that TOPORS (i) directly associates with RAD51, (ii) is phosphorylated by ATM on Thr515, (iii) enhances RAD51 recruitment to DSBs, (iv) induces SUMOylation of RAD51, (v) promotes HR but not NHEJ repair of DSBs, (vi) itself is recruited to DSBs and (vii) contributes to the maintenance of chromosome stability. We further identified two SUMO modification sites on RAD51 (K57 and K70), and the modification-deficient RAD51 mutant is less capable of supporting the normal HR-regulating activities of RAD51, convincingly arguing the physiological importance of the SUMO modification of RAD51.

A number of studies previously showed that the SUMO conjugation system is important during DSB repairs. The primary SUMO E3 ligases involved are PIAS1 and PIAS4, which themselves localize to laser microirradiation-induced DSBs, to promote DSB repair in part by inducing SUMOylation of 53BP1 (43) and BRCA1 (43,50). PIAS4 is also required for proper regulation of the HR repair, through SUMOylating the DSB end resection nuclease CtIP (51,52). Consistently, PIAS4 is required for RAD51 foci formation (35); this study did not find RAD51 itself to be SUMOylated, but a SIM in RAD51 is required for HR repair, suggesting that a noncovalent SIM–SUMO interaction is involved in regulating the HR repair. A study further found that the noncovalent interaction between the SIM of RAD51 and the SUMO-modified BLM helicase is necessary for efficient RAD51 foci formation at DSBs (27). While the primary mechanism by which PIAS4 is known to regulate the HR repair is through CtIP SUMOylation, our unbiased identification of TOPORS as a RAD51 regulator and extensive analysis of RAD51 SUMOylation show that a previously unknown SUMO-dependent RAD51 regulation exists. Whether a functional crosstalk between PIAS4 and TOPORS exists during the HR repair remains to be investigated in future, although our analysis did not suggest that PIAS4 is a direct regulator of RAD51.

How does SUMOylation regulate the RAD51’s activity? Interestingly, we found that the SUMOylation-deficient RAD51 is less capable in associating with BRCA2. As a primary function of BRCA2 is to load RAD51 onto ssDNA generated at DSB ends, we surprisingly found a new mechanism of SUMO during the crucial event during HR repair. Whether SUMOylation enhances RAD51 filament formation onto ssDNA would be interesting to address in future work, although such effect is likely based on the crucial role of BRCA2 in RAD51 loading on ssDNA. Consistently, the SUMO-deficient RAD51 mutant is less capable in supporting the RAD51 foci formation and the reporter-based HR repair activity, two common readouts used for assessing the HR repair. Importantly, the SUMO-deficient mutant is less capable in BRCA2 interaction. How SUMOylation of RAD51 promotes the BRCA2 interaction remains to be investigated; it is interesting to speculate that an intramolecular interaction takes place between the SUMO moiety and the SIM within the RAD51 protein, which may affect the BRCA2 interaction. Alternatively, the BRCA2 protein may harbor a cryptic SIM that is currently unrecognized. The SUMO-modified RAD51 might also engage in interacting with other SIMs in surrounding proteins, such as one present in RAD51AP1 (53,54) or FANCI (55). Further investigation is needed to decipher the regulatory modes.

TOPORS was initially identified as a topoisomerase I- and p53-binding protein (p53BP3), and it is the first nuclear protein that functions as both a ubiquitin and a SUMO E3 ligase (40,46,56,57). It has been suggested that TOPORS is a tumor suppressor, and that its expression is reduced or undetectable in malignancies (58). The tumor suppressor function of TOPORS is attributed to its ability to PTM anti-oncogenic proteins, including p53 (40,46) and mSin3A (42). TOPORS-deficient mice exhibit an increased rate of malignant transformation that is associated with aneuploidy and defective chromosomal segregation (36). While TOPORS has been previously suggested to be involved in base excision repair (59), not much is known about its direct substrates during DDR or DSB repair. Our work unequivocally shows that TOPORS is a new regulator of HR repair through directly SUMOylating RAD51. Unlike that PIAS4 is required for both branches of DSB repair, we found that TOPORS preferentially affects the HR activity, as knockdown of TOPORS did not affect NHEJ activity. Of note, a TOPORS knockdown has been shown to result in suppression of the pro-apoptotic p53 response because it is normally a positive regulator of p53 in response to DNA damage (46,60). In the present study, we showed that TOPORS-depleted cells exhibit increased sensitivity to IR. Therefore, the ability of TOPORS to promote HR repair may override its pro-apoptotic function.

In summary, our work identified a new role of SUMO and TOPORS in the HR repair; upon DNA damage, ATM-mediated phosphorylation of TOPORS interacts with RAD51 and SUMOylates RAD51. SUMOylated RAD51 is then efficiently recruited to sites of DNA breaks, likely triggered through its association with BRCA2, and subsequently promotes HR. In the absence of TOPORS, recruitment of RAD51 to DNA lesions is reduced, leading to lower levels of HR. Given the essential role of RAD51 in the error-free repair of DSBs, it is evident that the TOPORS-induced RAD51 SUMOylation is critical for the preservation of genome integrity (Figure 6E).

## Supplementary Material

gkac009_Supplemental_File
