# Supplementary material for: TOPORS-mediated RAD51 SUMOylation facilitates homologous recombination repair
Source: Nucleic Acids Res. 2022 Jan 21;50(3):1501–16. doi: 10.1093/nar/gkac009 (PMC8860612; doi:10.1093/nar/gkac009)
Supplement: gkac009_Supplemental_File [file gkac009_supplemental_file.docx]

**TOPORS-mediated RAD51 SUMOylation facilitates homologous recombination repair**

7 Supplementary Figures

3 Supplementary Tables

**
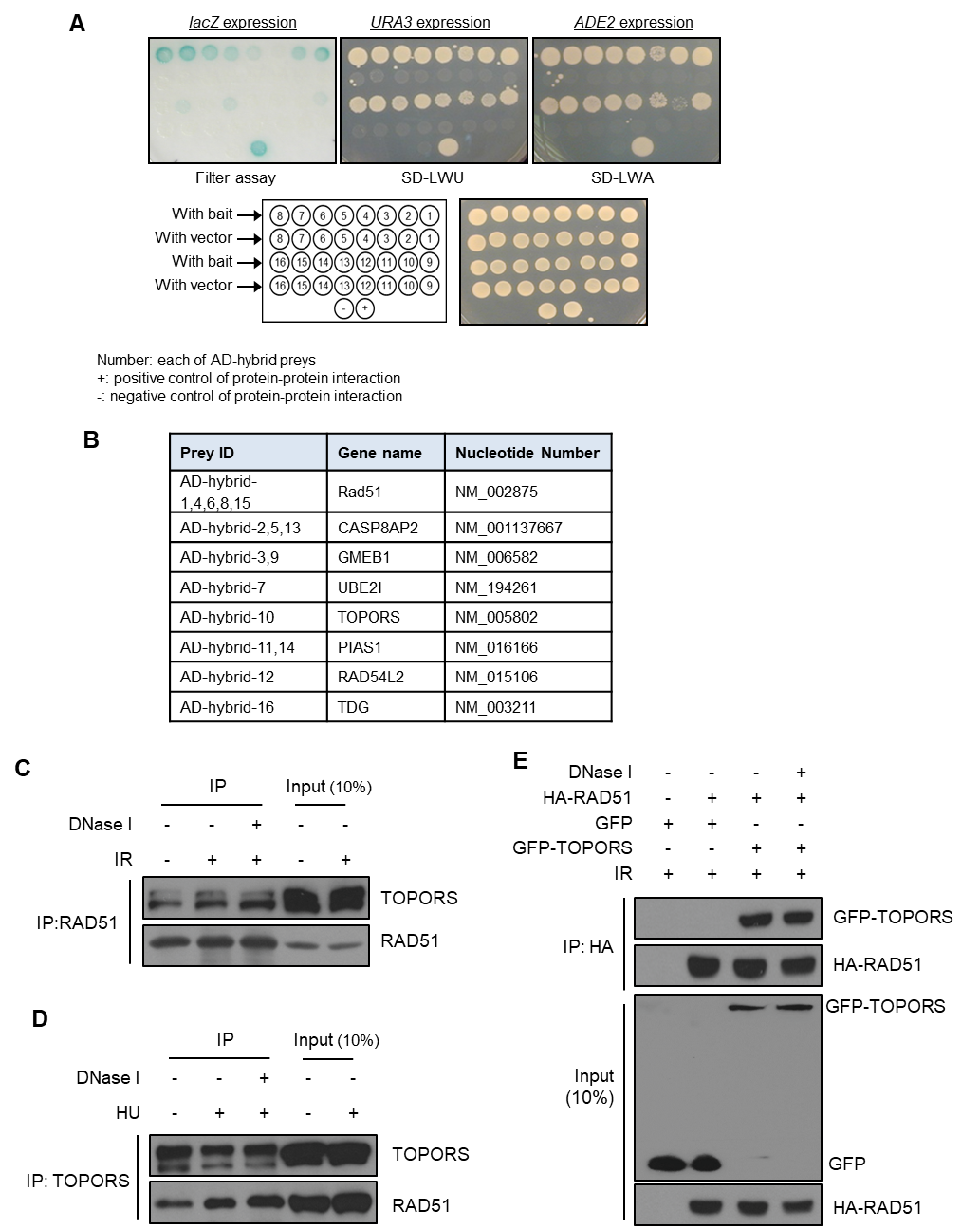
**

**
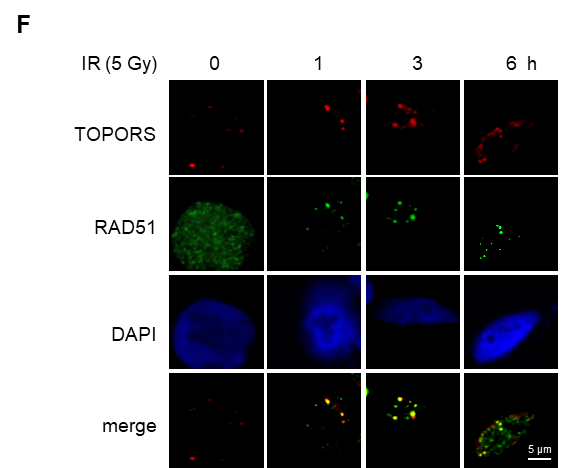
**

**Figure S1. RAD51 interacts with TOPORS.**

**(A)** Yeast two-hybrid screening identifies proteins that interact with the full-length RAD51. Yeast cells were transformed with a bait plasmid expressing either a GAL4-DNA binding domain (BD) (pGBKT7) or a GAL4-BD-fused full-length of RAD51 and a prey plasmid expressing a GAL4 transcription activation domain-fused HeLa cDNA library. Transformed yeast cells were selected on minimal media lacking leucine and tryptophan (SD-LW) to select for bait and prey plasmids, respectively. Left: Specific interactions between two proteins was monitored by the appearance of visible blue color on a filter assay; middle: the growth of colonies on selective medium lacking leucine, tryptophan and uracil (SD-LWU); right: the growth of colonies on selective medium lacking leucine, tryptophan and adenine (SD-LWA). From this screening, 24 positive clones were identified. pGBKT7-53 (p53) and pGADT7-T (SV40 large T-antigen) served as a positive control for the protein-protein interaction. pGBKT7 and pGADT7 were used as the negative control. **(B)** A list of proteins identified in the screening that interacted with RAD51. The identity of each clone was determined by automated DNA sequencing and BLAST analysis. **(C, D)** The effect of DNase I on interactions between RAD51 and TOPORS was measured by immunoprecipitation assay. HeLa cells were exposed to 5 Gy of IR, and 3 h later, whole cell lysates were treated with 100 μg/ml DNase I at 37°C for 20 min, or were mock-treated. Cell lysates were then immunoprecipitated using the anti-RAD51 (**C**) or anti-TOPORS antibody (**D**) and detected by Western blotting using the antibodies indicated to the right of the blot. **(E)** HeLa cells transfected with the indicated combinations of expression vectors for HA-RAD51, GFP Mock, and GFP-TOPORS were exposed to 5 Gy of IR. Three hours later, whole cell lysates were treated with 100 μg/ml DNase I at 37°C for 20 min, or were mock-treated. Cell lysates were then subjected to immunoprecipitation using anti-HA and detected by Western blotting using the antibodies indicated to the right of the blot. **(F)** TOPORS colocalizes with RAD51. Confocal microscopy imaging to determine the subcellular distribution of TOPORS and RAD51 in HeLa cells exposed to 5 Gy of IR and fixed at the indicated time points. Immunostaining was performed using anti-RAD51 and anti-TOPORS antibodies. Colocalization of TOPORS (red) and RAD51 (green) is visible as a yellow merged signal. Nuclei were stained with DAPI. Scale bars: 5 μm.

**
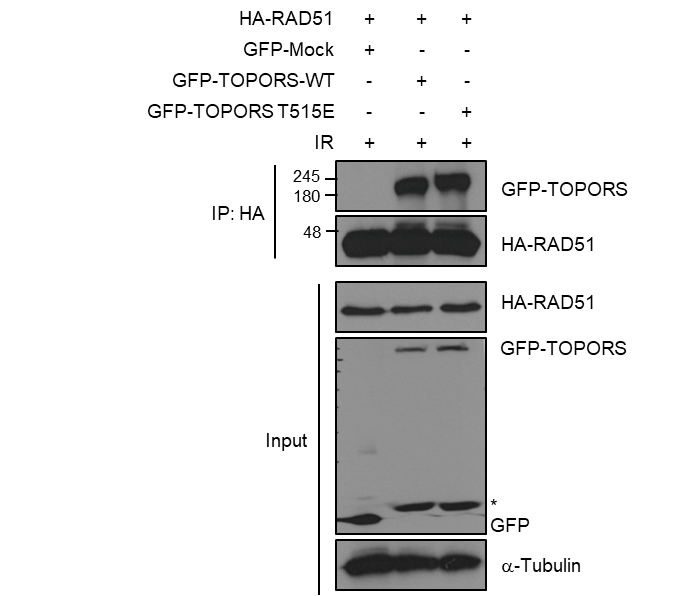
**

**Figure S2. RAD51 interacts with both WT and phosphomimetic (T515E) TOPORS in response to IR.**

HA-RAD51-expressing HEK293T cells transfected with control GFP vector, GFP-TOPORS-WT, or GFP-TOPORS-T515E were treated with 5 Gy of IR. Whole cell lysates were then subjected to immunoprecipitation with an anti-HA antibody followed by immunoblotting using indicated antibodies. Asterisk indicates degradation products of GFP-TOPORS.


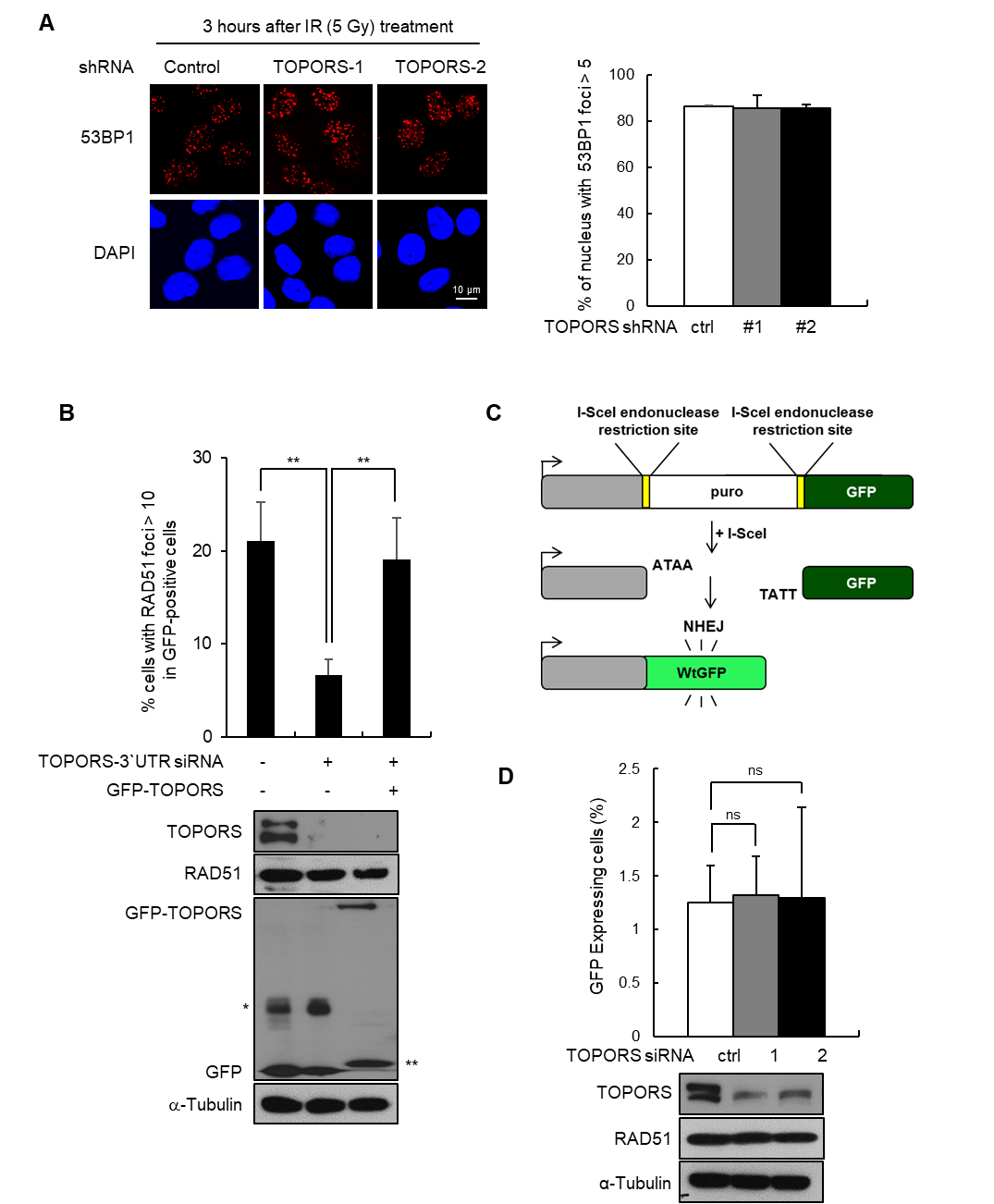


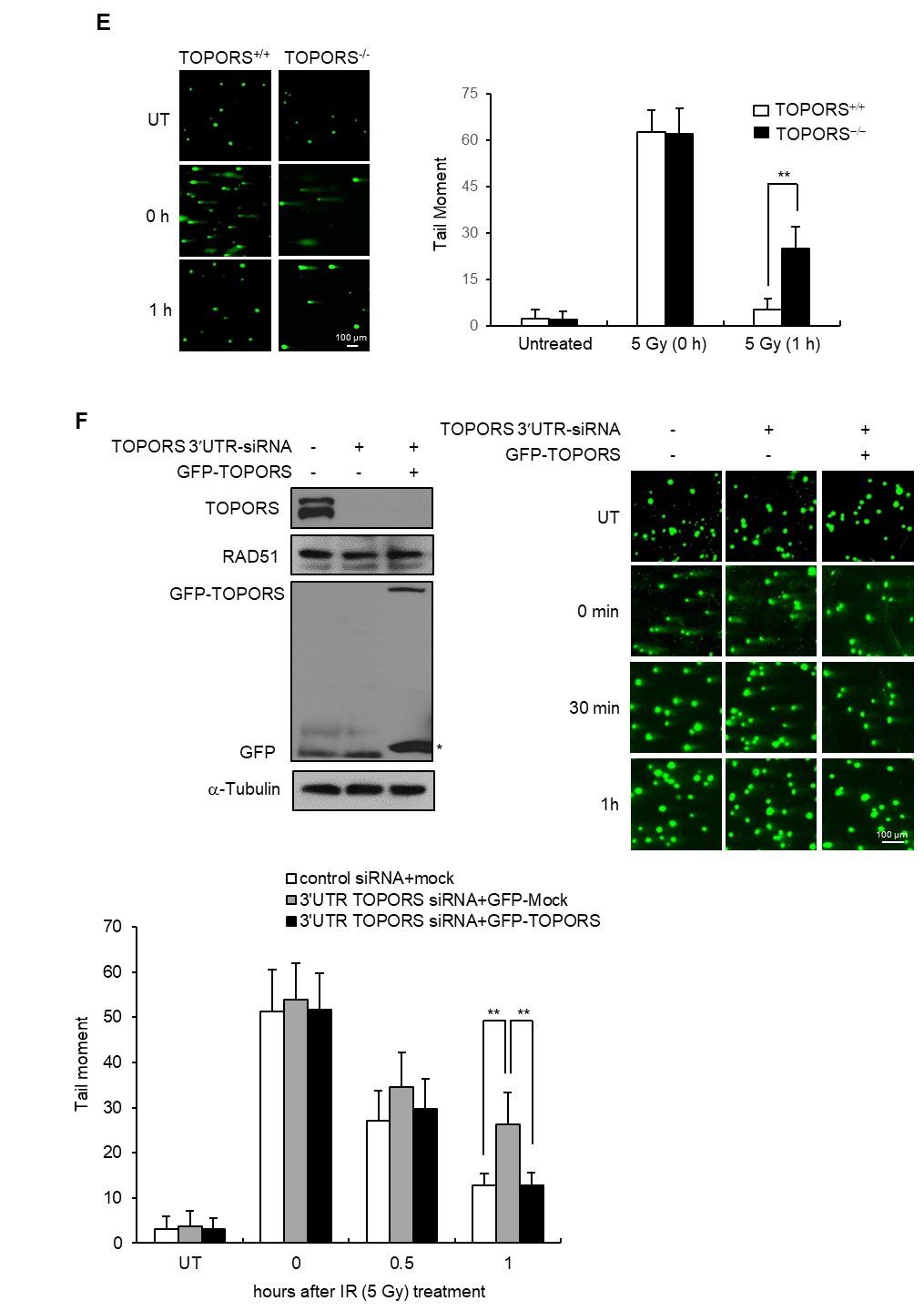


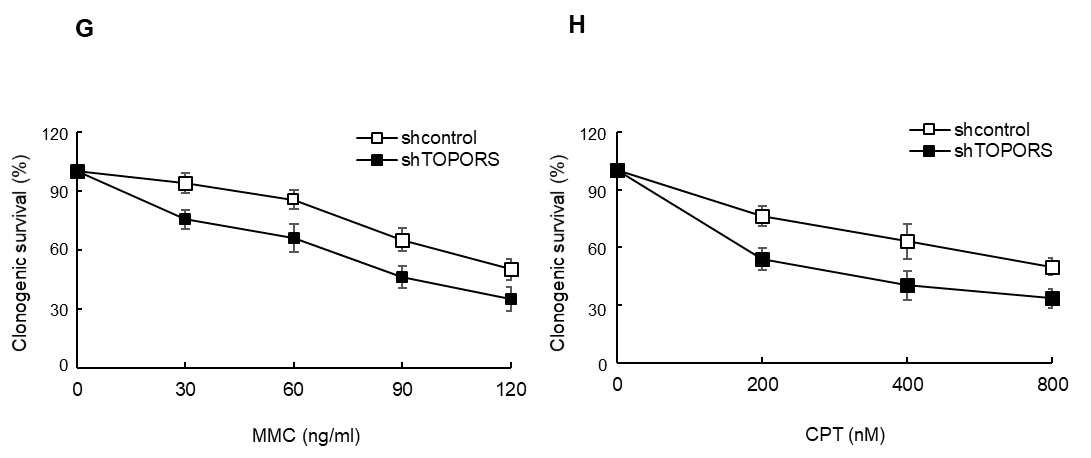


**Figure S3.** **TOPORS plays an important role in HR-mediated DSB repair.**

**(A)** Control- and TOPORS-depleted U2OS cells 5 Gy of IR and fixed at 3 h after IR exposure. Immunostaining experiments were performed using an anti-53BP1 antibody. DNA were counterstained with DAPI. Scale bar: 10 μm. Percentage of cell populations that shows more than 5 foci for 53BP1 is shown. The results are shown as mean ± SD (n = 3), ^**^*P* < 0.01. **(B)** Control and TOPORS knockdown HeLa cells reconstituted with GFP-Mock or GFP-TOPORS were treated with 5 Gy of IR, fixed at 3 h, and immunostained using an anti-RAD51 antibody. Nuclei were stained with DAPI. Scale bar: 10 μm. The percentage of cell populations that shows more than 10 foci for RAD51 in GFP-positive cells are shown. The results are shown as mean ± SD (n = 3), ** *P* < 0.01. One and two asterisks indicate nonspecific bands and degradation products of GFP-TOPORS, respectively. **(C)** A schematic of the assay used to measure non-homologous end joining (NHEJ) repair using an EJ5-GFP reporter construct. **(D)** Flow cytometry analysis to measure the efficiency of EJ5-GFP HeLa cells transfected with control or two different TOPORS siRNAs. Endogenous TOPORS and RAD51 levels in control and TOPORS siRNA-transfected cells were analyzed by western blotting. The results are shown as mean ± SD (n = 3). ns, not significant. **(E)** IR-induced DNA damage, as measured by a neutral comet assay, of control and Topors^−/−^ MEF cells treatment with or without 5 Gy of IR at the indicated time points. Representative images (left) and quantification (right) of the comet tail moments are shown. The results are shown as mean ± SD (n = 3), ** *P* < 0.01. Asterisk indicates nonspecific band. **(F)** DNA damage as measured by a neutral comet assay in control HeLa cells and TOPORS knockdown HeLa cells reconstituted with GFP-Mock or GFP-TOPORS after treatment with 5 Gy of IR at the indicated time points. Representative images (right) and quantification (bottom) of the comet tail moments are shown. The results are shown as mean ± SD (n = 3), ** *P* < 0.01. Asterisk indicates degradation products of GFP-TOPORS. **(G, H)** Colony forming ability of control- and TOPORS-depleted U2OS cells treated with the indicated doses of mitomycin C (MMC) (**G**) or camptothecin (CPT) (**H**). The results are shown as mean ± SD (n = 3), ^**^*P* < 0.01.

**
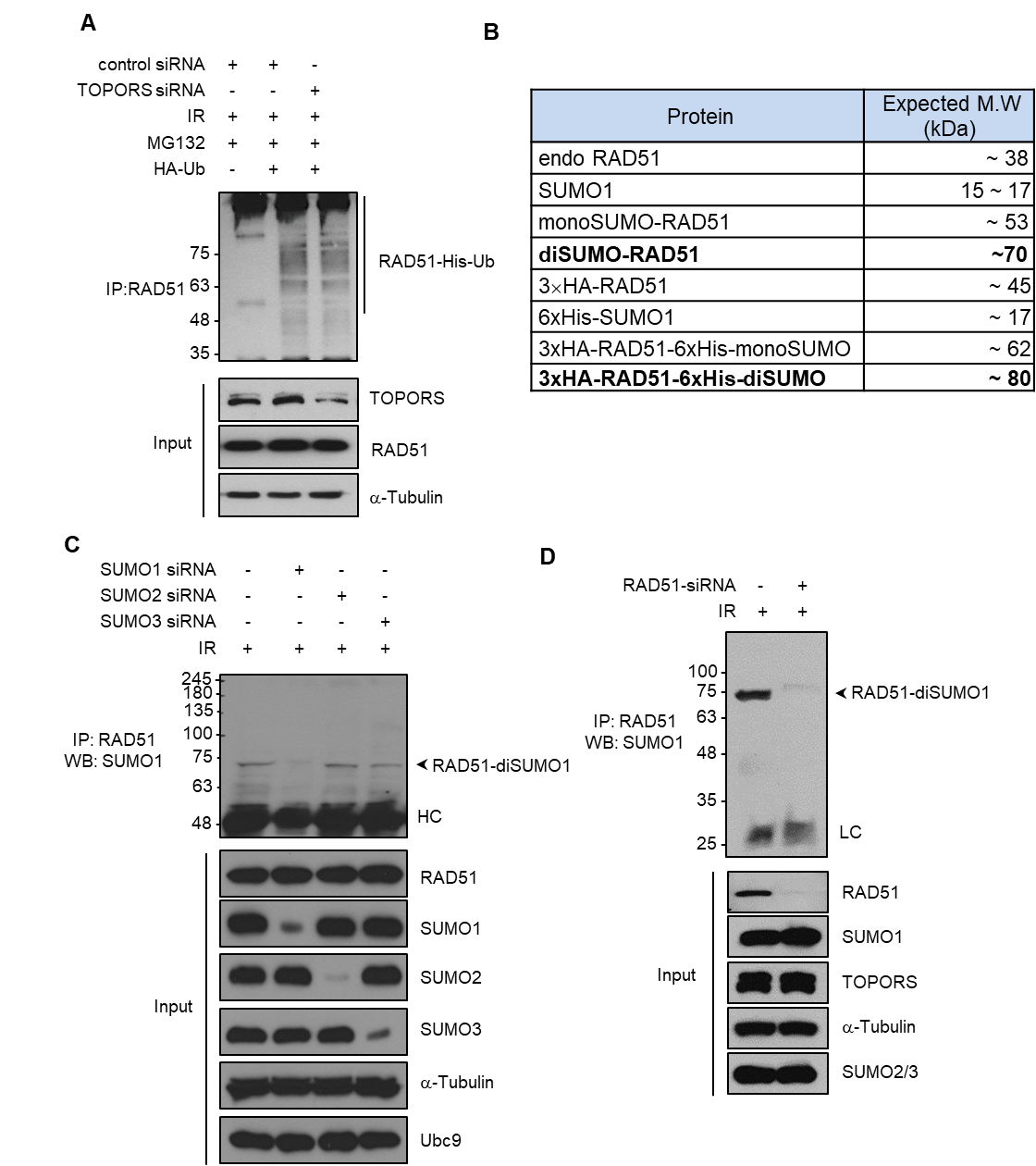
**

**Figure S4** **TOPORS SUMOylates RAD51 upon IR treatment.**

**(A)** HeLa cells were transfected with HA-Ub along with control or TOPORS siRNA, treated with 5 Gy of IR. 1 h after IR treatment, cells were treated with 10 μM MG132 for 4 h. Immunoprecipitations using the anti-RAD51 antibody were then performed and the following immunoblot analyses were done using anti- HA antibody. **(B)** List of observed M.W. of tagged and untagged Rad51, SUMO1 and SUMO1-RAD51 used in this study. **(C)** HeLa cells were transfected with control, SUMO1, SUMO2 or SUMO3 siRNA, irradiated with 5 Gy of IR, and immunoprecipitated with an anti-RAD51 antibody and immunoblotted with anti-SUMO1 antibody. HC indicates heavy chain. **(D)** HeLa cells transfected with control or RAD51 siRNA were irradiated with 5 Gy of IR, and subjected to immunoprecipitation followed by immunoblotting as indicated antibodies. LC indicates light chain.


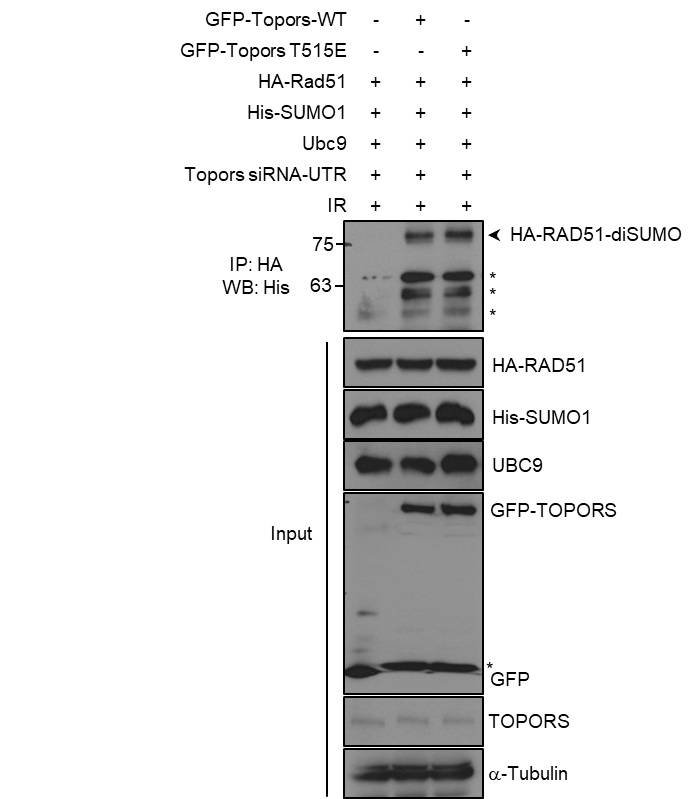


**Figure S5. The effect of phosphomimic mutant of TOPORS on RAD51 SUMOylation.**

TOPORS knockdown HEK293T cells were transfected with GFP-TOPORS WT or GFP-TOPORS (T515A) along with the indicated plasmids, exposed to 5 Gy of IR, and immunoprecipitated and immunoblotted as indicated. Asterisks indicate degradation products of GFP-TOPORS.

**
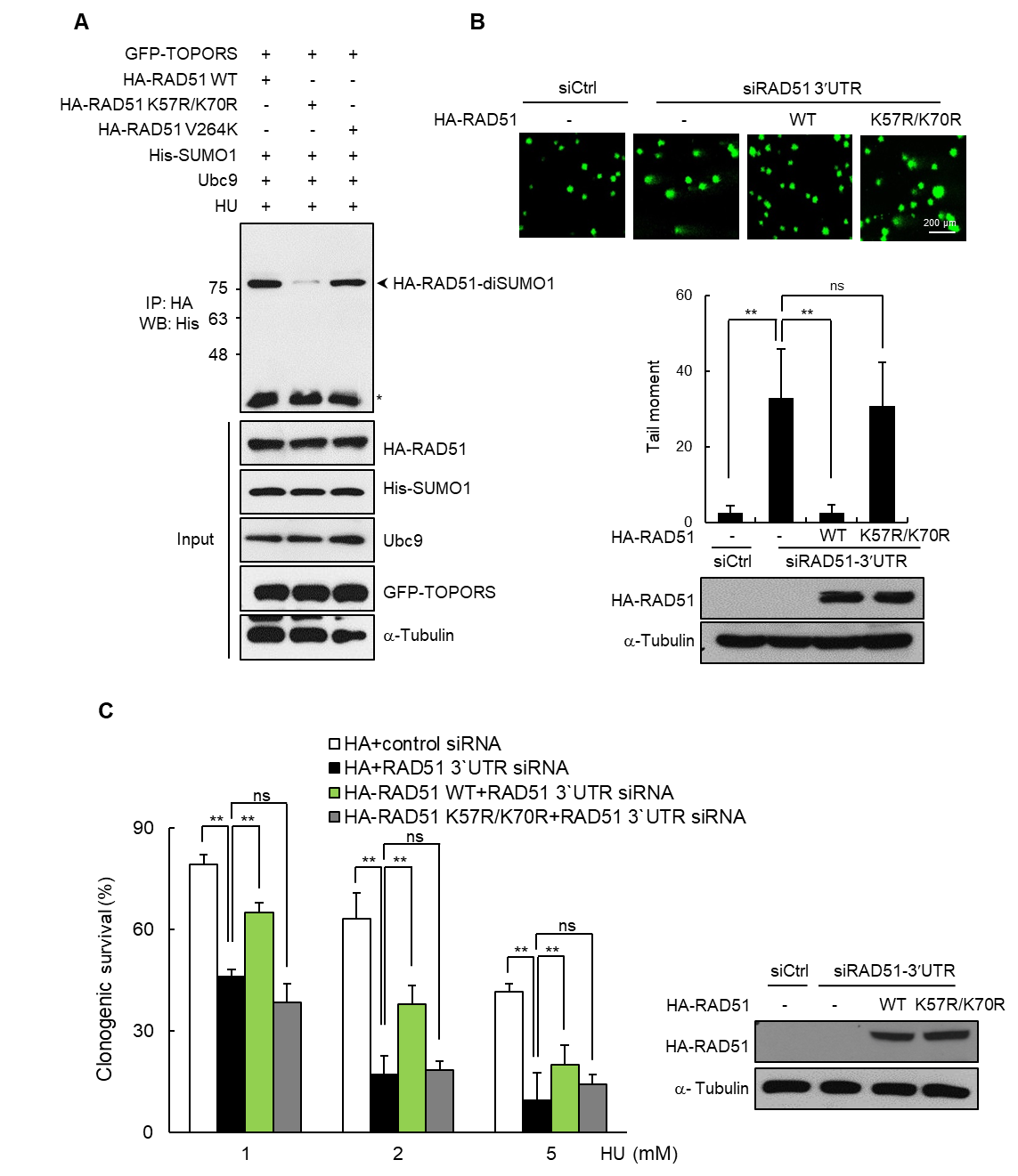
**

**
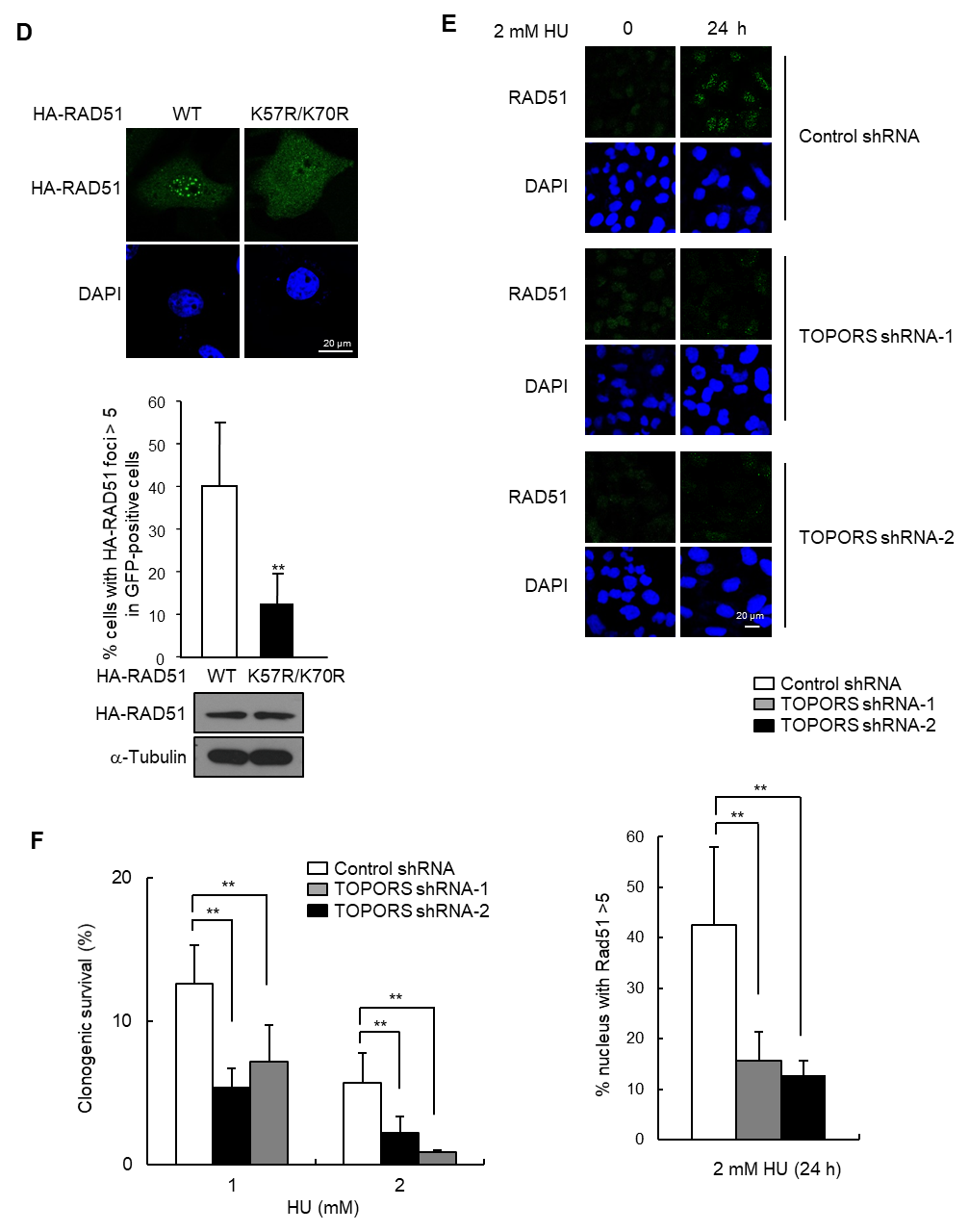
**

**Figure S6. TOPORS phosphorylation on Thr515 promotes RAD51-mediated DSB repair.**

**(A)** Immunoblotting with an anti-His antibody to detect SUMO1 from HA-RAD51 immunoprecipitated HEK293T cells transfected with HA-RAD51 WT or K57R/K70R together with the indicated plasmids and treated with 2 mM HU. Asterisk indicates nonspecific band. **(B)** HU-induced DNA damage as measured by a neutral comet assay in RAD51 knockdown HeLa cells reconstituted with HA-RAD51WT or HA-RAD51 K57R/K70R. Scale bar: 200 μm. The results are shown as mean ± SD (n = 3), ^**^*P* < 0.01. ns, not significant. **(C)** Colony forming ability of the same cells as described in (B). Cells were treated with the indicated doses of HU. The results are shown as mean ± SD (n = 3), ^**^*P* < 0.01. ns, not significant. **(D)** Quantification of HA-RAD51 foci in HeLa cells transfected with either HA-RAD51WT or HA-RAD51 K57R/K70R and treated with 2 mM HU. Immunostaining for HA was carried out 24 h after treatment with 2 mM HU. DNA were counterstained with DAPI. The percentage of cell populations that shows more than 10 foci for HA-RAD51 is shown. The results are shown as mean ± SD (n = 3), ^**^*P* < 0.01. **(E)** Control- and TOPORS-depleted U2OS cells were untreated or treated with 2 mM HU and fixed at the indicated time points. Immunostaining experiments were performed using an anti-RAD51 antibody. DNA were counterstained with DAPI. Scale bar: 10 μm. Percentage of cell populations that shows more than 5 foci for RAD51 is shown. The results are shown as mean ± SD (n = 3), ^**^*P* < 0.01. **(F)** Colony forming ability of control- and TOPORS-depleted U2OS cells treated with the indicated doses of HU. The results are shown as mean ± SD (n = 3), ^**^*P* < 0.01.

**
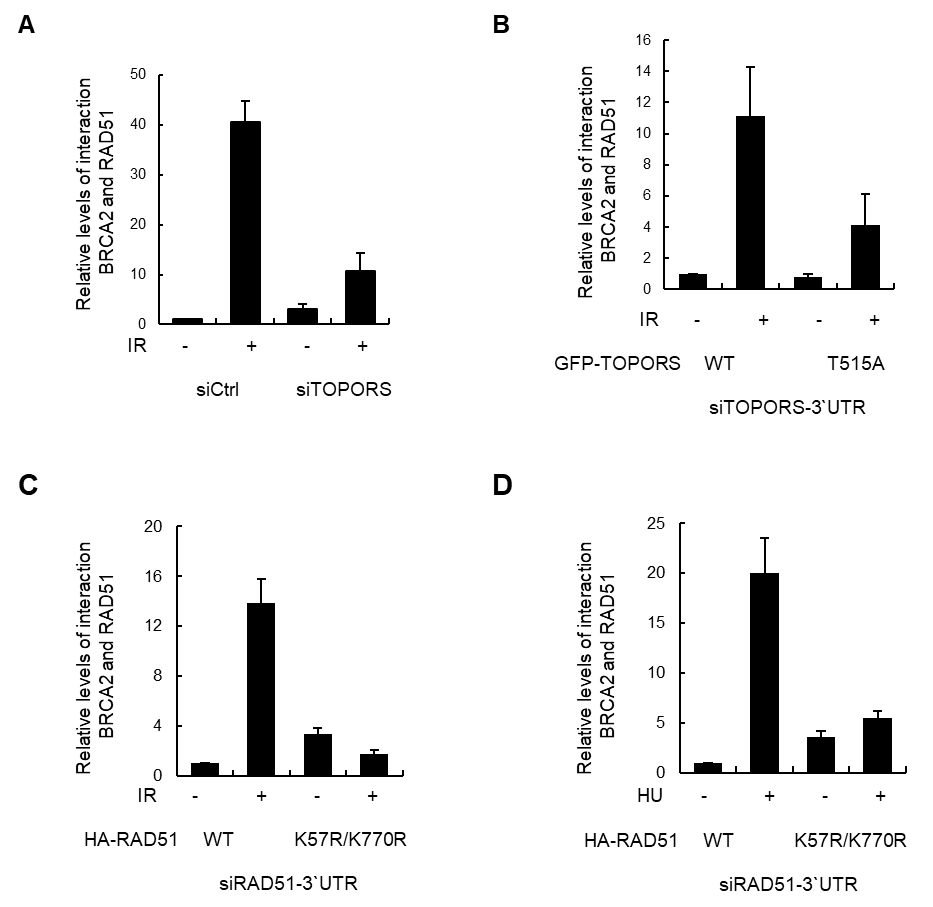
**

**Figure S7. Quantification of RAD51 and BRCA2 binding**

(A-D) Quantitative analysis of interaction between RAD51 and BRCA2 shown in Figure 6A-D. The results are shown as mean ± SD (n = 3).

**Table S1. List of antibodies used in this study: Western blot (WB), Immunoprecipitation (IP), Immunofluorescence (IF), and In situ Proximity Ligation Assay Immunohistochemistry (PLA).**

| **Antibody** | **Species** | **Application** | | | **Reference/Suppliers** | |
| --- | --- | --- | --- | --- | --- | --- |
|  |  | **WB** | **IF** | **IP** | **Suppliers** | **Cat. No** |
| TOPORS(5G11) | Mouse | 1:1000 | 1:200  1:100 (PLA) | 1:1000 | Abnova | H00010210-M01 |
| TOPORS | Mouse | 1:1000 |  |  | Santa cruz | sc-101182 |
| TOPORS | Rabbit | 1:1000 |  |  | Bethyl | A302-179A |
| Phospho-threonine | Mouse | 1:1000 |  |  | Sigma | P3555 |
| Phospho-threonine | Mouse | 1:1000 |  |  | Cell Signaling | 9386S |
| ATM | Mouse | 1:1000 |  |  | Rockland | 200-301-400 |
| RAD51 | Rabbit | 1:1000 | 1:200 | 1:1000 | Santa cruz | sc-8349 |
| RAD51 | Rabbit | 1:1000 | 1:100 (PLA) | 1:1000 | Abcam | ab63801 |
| RAD51(14B4) | Mouse | 1:1000 |  |  | Abcam | ab213 |
| RAD51 clone 3C10 | Mouse | 1:1000 |  |  | Millipore | 05-530 |
| HA | Mouse | 1:1000 | 1:200 | 1:1000 | Santa cruz | sc-7392 |
| HA | Rabbit | 1:1000 |  | 1:1000 | Santa cruz | sc-805 |
| HA | Mouse | 1:1000 |  | 1:1000 | Abcam | ab18181 |
| HA | Rabbit |  | 1:100 | 1:1000 | Cell signaling | C29F4 |
| GFP | Mouse | 1:1000 |  | 1:1000 | Santa cruz | sc-9996 |
| GFP | Rabbit | 1:1000 |  |  | Santa cruz | sc-8334 |
| GFP | Rabbit |  |  | 1:1000 | Novus | NB600-308 |
| His | Mouse | 1:1000 |  | 1:1000 | Santa cruz | sc-8036 |
| His | Rabbit | 1:1000 |  | 1:1000 | Santa cruz | sc-804 |
| His | Rabbit | 1:1000 |  |  | Cell signaling | #2365 |
| His | Mouse | 1:1000 |  |  | Abcam | ab18184 |
| FLAG | Mouse | 1:1000 |  |  | AbFrontier | MA-20168 |
| FLAG | Rabbit |  |  | 1:1000 | Sigma | F7425 |
| GST(B-14) | Mouse | 1:1000 |  |  | Santa cruz | sc-138 |
| γ-H2AX (Ser139) | Mouse | 1:1000 | 1:200 |  | Millipore | 05-636-1 |
| PML | Mouse | 1:1000 |  |  | Santa cruz | sc-966 |
| PML(N-19) | Goat |  | 1:200 |  | Santa cruz | sc-9862 |
| SUMO1 | Rabbit | 1:1000 |  |  | Abcam | ab32058 |
| SUMO1/GMP-1 | Mouse | 1:1000 |  |  | Invitrogen | REF332400 |
| SUMO2/3 (sentrin-2) | Rabbit | 1:1000 |  |  | Invitrogen | REF519100 |
| Ubc9 | Goat | 1:1000 |  |  | Abcam | ab21193 |
| α-Tubulin | Mouse | 1:1000 |  |  | Santa cruz | sc-5286 |
| β-actin | Mouse | 1:1000 |  |  | Santa cruz | sc-47778 |
| SENP2 | Rabbit | 1:1000 |  |  | abcam | ab3660 |
| BRCA2 | Rabbit | 1:1000 |  |  | Bethyl | A303-434A |
| BRCA2 | Rabbit | 1:1000 |  |  | abcam | ab123491 |
| BRCA2 (Ab-1) | Mouse | 1:1000 |  |  | Calbiochem | OP95 |
| Ubiqutin (P4D1) | Mouse | 1:1000 |  |  | Santa cruz | sc-8017 |
| Duo link In situ PLA Probe | Rabbit Minus |  | 1:200(PLA) |  | Sigma | DUO92005 |
| Duo link In situ PLA Probe | Mouse Plus |  | 1:200(PLA) |  | Sigma | DUO92001 |
| Sheep Anti-Mouse IgG | sheep | 1:4000 |  |  | Jackson immunoresearch | 515-035-071 |
| Donky Anti-Goat IgG | donky | 1:4000 |  |  | Jackson immunoresearch | 705-035-003 |
| Donky Anti-Rabbit IgG |  | 1:4000 |  |  | Jackson immunoresearch | 711-035-152 |
| Donky Anti-Mouse IgG |  | 1:4000 |  |  | Jackson immunoresearch | 715-035-150 |
| Alexa Fluor 488 Chicken Anti-Rabbit IgG(H+L) | chicken |  | 1:200 |  | Invitrogen | A-21441 |
| Alexa Fluor 488 Chicken Anti-Mouse IgG(H+L) | chicken |  | 1:200 |  | Invitrogen | A-21200 |
| Alexa Fluor 594 Chicken Anti-Goat IgG(H+L) | chicken |  | 1:200 |  | Invitrogen | A-21468 |
| Alexa Fluor 594 Chicken Anti-Mouse IgG(H+L) | chicken |  | 1:200 |  | Invitrogen | A-21201 |
| Alexa Fluor 594 Chicken Anti-Rabbit IgG(H+L) | chicken |  | 1:200 |  | Invitrogen | A-21442 |
| Alexa Fluor 647 Chicken Anti-mouse IgG(H+L) | chicken |  | 1:200 |  | Invitrogen | A-21463 |

**Table S2. List of primer sequence used for cloning.**

| **Construct** | **Forward primer sequence** | **Reverse primer sequence** |
| --- | --- | --- |
| HA-RAD51-WT | gcctctagaatggcaatgcagatg | aatgggccctcagtctttggcatc |
| HA-RAD51-K57R | gttgcctatgcgccacggaaggagctaata | tattagctccttccgtggcgcataggcaac |
| HA-RAD51-K70R | agtgaagcccgagctgataaaattctggctg | cagccagaattttatcagctcgggcttcact |
| pET28a-RAD51 | aacggatccatggcaatgcagatg | aatgtcgacagtctttggcatctcc |
| pGEX4T1-TOPORS | aaagaattcatggggtcgcagc | acccagctgagacatatcacagtc |
| pEGFP-N3-T515A | atggagacagtgaaggcacaagaacaggagcaa | ttgctcctgttcttgtgccttcactgtctccat |
| pEGFP-N3-T515E | atggagacagtgaaggaacaagaacaggagcaa | ttgctcctgttcttgttccttcactgtctccat |
| HA-Rad51 V264K | tggtgtagcagtgaaaaycactaatcaggtgg | ccacctgattagtgattttcactgctacacca |

**Table S3. The raw data for Figure 3D and 3E.**

| **Fig 3D** | control  siRNA | TOPORS  siRNA #1 | TOPORS  siRNA #2 |
| --- | --- | --- | --- |
| set1 | 5.96 | 2.74 | 3.39 |
| set2 | 5.93 | 3.55 | 3.33 |
| set3 | 5.83 | 2.51 | 3.26 |
| average | 5.91 | 2.93 | 3.33 |

| **Fig 3E** | sicontrol | siTOPORS | siRAD51 | siTOPORS + siRad51 |
| --- | --- | --- | --- | --- |
| set1 | 6.32 | 3.08 | 0.93 | 2.36 |
| set2 | 5.73 | 2.94 | 1.87 | 2.18 |
| set3 | 5.99 | 1.21 | 1.96 | 2.19 |
| average | 6.01 | 2.41 | 1.59 | 2.24 |
